# The roles of DNA methylation on pH dependent i-motif (iM) formation in rice

**DOI:** 10.1093/nar/gkad1245

**Published:** 2024-01-05

**Authors:** Yilong Feng, Xing Ma, Ying Yang, Shentong Tao, Asgar Ahmed, Zhiyun Gong, Xuejiao Cheng, Wenli Zhang

**Affiliations:** State Key Laboratory of Crop Genetics and Germplasm Enhancement and Utilization, CIC-MCP, Nanjing Agricultural University, No.1 Weigang, Nanjing, Jiangsu 210095, China; State Key Laboratory of Crop Genetics and Germplasm Enhancement and Utilization, CIC-MCP, Nanjing Agricultural University, No.1 Weigang, Nanjing, Jiangsu 210095, China; State Key Laboratory of Crop Genetics and Germplasm Enhancement and Utilization, CIC-MCP, Nanjing Agricultural University, No.1 Weigang, Nanjing, Jiangsu 210095, China; State Key Laboratory of Crop Genetics and Germplasm Enhancement and Utilization, CIC-MCP, Nanjing Agricultural University, No.1 Weigang, Nanjing, Jiangsu 210095, China; State Key Laboratory of Crop Genetics and Germplasm Enhancement and Utilization, CIC-MCP, Nanjing Agricultural University, No.1 Weigang, Nanjing, Jiangsu 210095, China; Bangladesh Wheat and Maize Research Institute (BWMRI), Nashipur, Dinajpur 5200, Bangladesh; Jiangsu Key Laboratory of Crop Genomics and Molecular Breeding, Agricultural College of Yangzhou University, Yangzhou 225009, China; State Key Laboratory of Crop Genetics and Germplasm Enhancement and Utilization, CIC-MCP, Nanjing Agricultural University, No.1 Weigang, Nanjing, Jiangsu 210095, China; State Key Laboratory of Crop Genetics and Germplasm Enhancement and Utilization, CIC-MCP, Nanjing Agricultural University, No.1 Weigang, Nanjing, Jiangsu 210095, China

## Abstract

I-motifs (iMs) are four-stranded non-B DNA structures containing C-rich DNA sequences. The formation of iMs is sensitive to pH conditions and DNA methylation, although the extent of which is still unknown in both humans and plants. To investigate this, we here conducted iMab antibody-based immunoprecipitation and sequencing (iM-IP-seq) along with bisulfite sequencing using CK (original genomic DNA without methylation-related treatments) and hypermethylated or demethylated DNA at both pH 5.5 and 7.0 in rice, establishing a link between pH, DNA methylation and iM formation on a genome-wide scale. We found that iMs folded at pH 7.0 displayed higher methylation levels than those formed at pH 5.5. DNA demethylation and hypermethylation differently influenced iM formation at pH 7.0 and 5.5. Importantly, CG hypo-DMRs (differentially methylated regions) and CHH (H = A, C and T) hyper-DMRs alone or coordinated with CG/CHG hyper-DMRs may play determinant roles in the regulation of pH dependent iM formation. Thus, our study shows that the nature of DNA sequences alone or combined with their methylation status plays critical roles in determining pH-dependent formation of iMs. It therefore deepens the understanding of the pH and methylation dependent modulation of iM formation, which has important biological implications and practical applications.

## Graphical Abstract

**Graphical Abstract:**
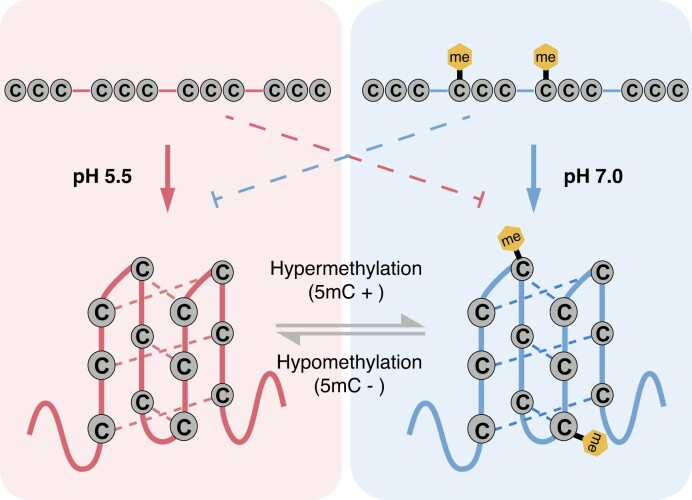

## Introduction

An i-motif (iM) is a type of quadruplex (four-stranded) non-B DNA structures also termed an i-tetraplex or i-DNA. iMs fold from C-rich DNA sequences and the resulting structures are stabilized by the formation of hemiprotonated C:C^+^ base-pairs, which form the steps of the resulting antiparallel duplex ladders. iMs thus fold preferentially under acidic conditions (1–3). The DNA hexamer 5′-d(TCCCCC) was first reported to form iM structures at acidic pH in 1993, whose structure was confirmed by NMR (1). iMs were initially assumed to be sensitive to pH and/or temperature (*T*_m_) with a decrease in their stability as an increase of pH and/or Tm values (4,5). Due to their acidic pH-dependency, iMs were originally considered as exotic structures, without consideration of their biological relevance under physiological conditions. Therefore, characterizations of iMs under normal cellular conditions were largely neglected as compared to their guanine (G) counterparts, the G-quadruplexes (or G4s), another non-B DNA structure that folds from G-rich sequences complementary with iM-forming sequences, whose biological relevance is thoroughly studied and now firmly established.

Accumulating evidence now shows that many parameters affect the formation and stability of iMs (6), including pH conditions (6), the nature of C-rich sequences, such as the number of Cs in the C runs (3,7) and the composition of the intervening sequences in between the C runs (8). For instance, longer loops promote the stability of iMs under neutral conditions, while shorter loops favor iM folded under acidic conditions (9), with the first two loops having less impacts on iM stability as compared to the other loops (10). Other factors display a significant influence, such as negative superhelicity (11) and epigenetic modifications. For instance, DNA oligoes with thymine replaced by 5-propynyluracil facilitate iM formation (12); 5mC and 5-bromocytosine (5-BrC) help stabilize iM formation at basic and acidic conditions (13), respectively, while 5hmC destabilizes iM formation (5,7,14,15). External factors could also affect iM stability, such as low temperature (16), molecular crowding (5,17,18); pressure (19); cationic conditions (7,20–23). Finally, the use of small interacting ligands has also been studied to modulate iM formation, for instance, TMPyP4 (1-methylpyridinium-4-yl) porphyrin) stabilizes iMs at pH 4.5 (24–26); BisA (a macrocycle containing two acridine subunits linked by two diethylenetriamine arms) stabilizes iMs at pH 6.8 (27); IMC-48 (small molecules for binding of iMs) and IMC-76 (small molecules for binding of the flexible hairpin) were studied for iM stabilization and destabilization, respectively (28,29); nanomaterials like carboxyl-modified single-walled carbon nanotubes(SWNTs) (30) and carboxyl-modified graphene quantum dots (GQDs) (31) can also affect iM formation. Further studies pinpoint complex effects of cytosine modifications on iM stability in humans and plants, which depend on the environmental conditions, the number and position of methylcytosines (5,15,32–34). Collectively, many factors are known and can be leveraged to fine-tune iM formation under neutral or alkaline conditions.

Growing evidence shows that iMs can also be involved in important biological processes in eukaryotic genomes (6,35–38), including gene transcription (39,40), especially for human oncogenes (8,35,41) and DNA replication (40,42), along with centromere and telomere activities (43–46), transposable element (TE) activities (32) and human pathogenesis (47). iMab antibody-based immunodetection has provided direct evidence about the cell cycle-dependent formation of iMs in human cells (48). Also, global profiling of iMs has been achieved in rice by iM-IP-seq (32) and human genomes by iM-ChIP-seq (40) or CUT&TAG (49). Thus, iM formation and its potential biological implications have attracted more attention recently, reaching a turning point towards comprehension and characterization of iMs in both plants and humans.

However, effects of pH (5.5 versus 7.0) on iM formation on a genome-wide scale are still completely uncharacterized in mammalians and plants. It has been documented that 5mC has modest impacts on the stability of iMs (50). CpG methylation could stabilize iM structures in neutral pH conditions (51–53), or induce pH dependent changes of iMs (54), further suggesting complex effects of DNA methylation on iM formation. Therefore, how DNA methylation affects iM formation on a genome-wide scale at pH 5.5 *versus* 7.0 is an intriguing question, which is also largely unknown in eukaryotes. To address this, we performed both iM-IP-seq and bisulfite sequencing (BS-seq) at pH 5.5 and 7.0 for comprehensive characterization of the influence of both pH and DNA methylation on iM formation in the rice genome.

## Materials and methods

### Plant materials

After pre-germination at room temperature (RT) for 3 days, uniformly germinated Nipponbare (*Oryza sativa* L., Japonica) rice seeds were put on the soil and grew in a greenhouse at 28–30°C and a 14 h/10 h light–dark cycle. Two-week old seedlings were collected and stored at –80°C until used. For pH 5.5 and 7.0 related transcriptomic assays, uniformly germinated rice seeds were grown in water for 5 days following by continuing to grow under pH 5.5 and pH 7.0, respectively, for additional 7 days. Rice seedlings under each pH conditions were collected for RNA-seq and CUT&TAG experiments.

For zebularine treatment, an inhibitor of DNA methyltransferase (55,56), 100 of 5-day-old rice seedlings were treated with 80 μM zebularine (Sigma-Aldrich Z4775) dissolved in DMSO or DMSO only (as control) for another 5 days. The zebularine-treated or untreated seedlings were collected and kept at –80°C until used.

For genomic DNA with M.CviP I (M0227S, NEB) treatment, a type of CpG methyltransferase resulting in a global increase of CG methylation (57,58). 4 μg of purified rice genomic DNA was incubated with 8 units of M.CviP I and an addition of 640 μM SAM (S-adenosyl-methionine) at 37°C for 1 h in a 20 μl reaction system. After inactivation of the enzyme activity with incubation at 65°C for 20 min, M.CviP I treated DNA was recovered by using phenol/chloroform extraction followed by pre-cold alcohol precipitation.

### iM-IP-seq and data analyses

iM-IP-seq was conducted as our previous procedures (32) using CK (a control with original genomic DNA without changes of DNA methylation) and hyper or de-methylated genomic DNA under Tris-AcOH buffer with pH 5.5 and 7.0 conditions. iMab/IgG-IPed and input DNA were isolated by using phenol/chloroform extraction followed by pre-cold alcohol precipitation. All libraries were prepared by using the NEBNext^®^ Ultra™ II DNA Library Prep Kit for Illumina (NEB, E7645S) and sequenced on Illumina NovaSeq platform. Raw iM-IP-seq data were trimmed by using Fastp to remove adapter sequences. All clean reads from two biological replicates were aligned to the MSU v7.0 reference genome (http://rice.plantbiology.msu.edu/ pub/data/Eukaryotic Projects/o sativa/annotation dbs/pseudomolecules/version 7.0/all.dir/) by using BWA (Burrows-Wheeler Aligner) (mem algorithm, version 0.7.17) (59) with default parameters. Reads with mapping quality below 10 were excluded by using SAMtools. PCR duplicates were removed by using Picard. The spearman rank correlation coefficient between replicates was calculated using multiBamSummary and plotCorrelation functions of deepTools (60). All aligned reads with at least length of 50 bp were used for peak calling by using callpeak functions of MACS2 (version 2.1.1) (61) with parameters as below: –extsize 200 -q 0.05 -nomodel -f BAMPE. Input and IgG data were used as controls.

### Differential peak analyses

pH biased and unbiased (common) peaks were identified by using MAnorm (v1.3.0) (62) with cutoffs as below: |*M*-value| > 0.5 and *P*-value < 0.001. *A*-value (*A* = 0.5 × log_2_ (Read density in sample 1 × Read density in sample 2)>) and *M*-value (*M* = log_2_ (Read density in sample 1/Read density in sample 2)) were calculated according to the normalized read counts within peaks.

### iM-IP-qPCR assay

1 μl of input and IPed DNA (2 ng/μl) was used as DNA template. pH 5.5 and 7.0 biased peaks were randomly selected for designing qPCR primers. The enrichment level of IPed DNA was calculated using the 2^(ΔΔCt)^ method and expressed as fold changes over the corresponding input. Each primer set was repeated three times in each qPCR. All primer sequences are listed in Supplementary Table S5.xlsx. Significance test was determined by using one-way ANOVA analysis, ***P < 0.001, **P < 0.01 and *P < 0.05.

### iMab based CUT&TAG-qPCR assay

Nuclei were purified from rice seedlings grown under pH 5.5 and 7.0 and incubated with ConA and iMab antibody. CTU&TAG was conducted by following the manual of the kit (Cat#: TD904-01, Vazyme) purchased from Vazyme. iMab antibody bound DNA was recovered for qPCR assay using primers from pH 5.5 and 7.0 related differentially expressed genes (DEGs) containing PiMFSs (putative i-motif forming sequences). Enrichment levels of IPed DNA were calculated using the 2^(ΔΔCt)^ method and expressed as fold changes over the corresponding input. Each primer set was repeated three times in each qPCR. All qPCR primer sequences are listed in Supplementary Table S5.xlsx. Significance test was determined by using one-way ANOVA analysis, ***P < 0.001, **P < 0.01 and *P < 0.05.

### RNA-seq and GO term enrichment analyses

Total RNA was extracted from seedlings grown under pH 5.5 and 7.0 conditions, respectively, for RNA-seq library preparation and sequencing, which was conducted by Berrygenomics company (Beijing). RNA-seq data were cleaned by removing adaptors and low-quality reads using the fastp v0.21.0 software (63) with default parameters. Clean reads were aligned to the rice reference genome (*Oryza sativa* L., MSU7) using HISAT2 v2.1.0 with default parameters (64) for calculation of read counts using featureCounts v1.6.4 (65). The fragments per kilobase per million mapped reads (FPKMs) and DEGs were identified using DESeq2 v1.36.0 package (66) in R (version 4.02). Gene Ontology (GO) enrichment analyses were conducted using the online database (AgriGO) specific for agricultural species (http://systemsbiology.cau.edu.cn/agriGOv2/index.php) with the threshold of *P*-value < 0.01 (67). All analyses were visualized using the ggplot2 package in R.

### Genomic distributions and calculation of GC content, GC/AT skew

Genomic distributions were investigated by using ChIPseeker (68) from the R package. To calculate GC content and GC/AT skew, ±1 kb regions around the center of PiMFSs-IP^+/−^ were divided into 50 bp windows, then were calculated using the formulas as below: GC content = (C + G)/(C + G + A + T); GC skew = (G – C)/(G + C) and AT skew = (A – T)/(A + T).

### Bisulfite-sequencing (BS-seq) and data analyses

5 μg of M. Cvip I-/zebularine-treated genomic and the corresponding CK DNA were used for bisulfite treatment followed by library preparation and sequencing, which was conducted by Berry Genomics company (Beijing). BS-seq data were trimmed by using Fastp to remove adapter sequences, then mapped to the rice reference genome (MSU7.0) by using Bismark (69). The methylated cytosines were counted from the total uniquely mapped reads using the Bismark methylation extractor script. DNA methylation levels were calculated using the total number of all (C + T) ≥ 3 in each position. DNA methylation levels across ±1 kb from the upstream to the downstream of different regions were profiled using 50 bp sliding windows. Methylation levels in each region were calculated using the formula: methylation level = the number of methylated cytosines/the total number of cytosines. Differentially methylated regions (DMRs), including hyper and hypo DMRs, were identified by using intersect and dmr functions of CGmapTools (70) with the cutoffs: *P* < 0.01 and ΔmC ≥0.1.

### Motif prediction

DNA sequences of common, pH 5.5 and 7.0 biased peaks were used for motif identification by using MEME-ChIP (http://memesuite.org/tools/meme-chip) with the parameters: minimum width 5 and maximum width 20. TF database of *Arabidopsis* was used to match putative TF-binding sites corresponding to all identified motifs by using Tomtom tool.

### Read count normalization

±1 kb from the upstream to the downstream of peaks and center of PiMFSs were divided into 50 bp windows. Each peak region was equally divided into 20 bins for counting normalized reads. The number of reads per sliding window was divided by the window length, then by the count of all unique reads within the genome. The midpoint of the fragment was used to determine the position in the rice genome.

### CD measurements

400 μl of 5 μM synthesized iM oligoes in C4 stabilization buffer (50 mM Tris-AcOH, pH 5.5 or 7.0) were measured in an optical chamber (1 mm path length) with a JASCO J-815 spectropolarimeter (Tokyo, Japan). Dry purified nitrogen was used to maintain a deoxygenation atmosphere. After subtracting the CD signal from the background solution, the final CD signal from iM oligoes examined was used to indicate iM folding potentials.

### Dot blotting assays

Synthesized DNA oligoes corresponding to pH 5.5/7.0 biased peaks, synthesized DNA oligoes with methylated C at different positions, genomic DNA, zebularine- and M.Cvip I-treated genomic DNA were denatured and re-associated in iM reconstruction buffer (50 mM Tris–AcOH, pH 5.5 or 7.0) at 95°C for 8 min. Denatured and reassociated DNA was loaded on Amersham Hybond-N^+^-nylon membrane followed by pre-blocking in 5% milk for 45 min at RT. The pre-blocked membrane was incubated with the iMab antibody in the standard blotting or genomic IP buffer with pH 5.5 or 7.0 overnight at 4°C, then incubated with anti-IgG (HRP) antibody for an additional 1.5 h. The procedures for immune-signal development were the same as our previous procedures (71). Each blot was repeated at least two times for signal quantification.

## Results

### *In vitro* identification of i-motifs at pH 7.0 in rice

**Figure 1.**
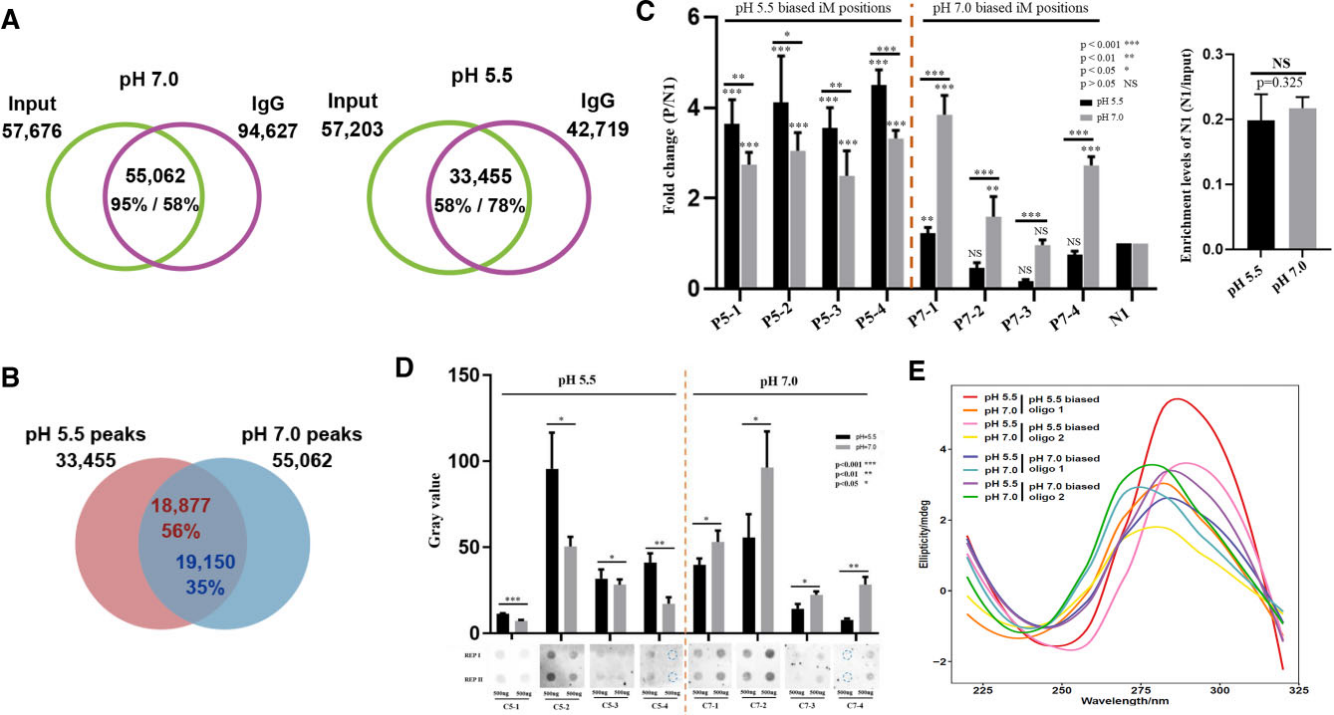
Identification and comparisons of *in vitro* iMs between pH 7.0 and 5.5 conditions. (**A**) Venn plots illustrating the number of iMs identified using iM-IP-seq relative to input or IgG as a control, corresponding to 55 062 and 33 455 common peaks at pH 7.0 and 5.5 conditions, respectively. (**B**) Venn plots illustrating overlaps of iMs between 55 062 at pH 7.0 and 33455 at pH 5.5, common peaks, pH 7.0 (*n* = 35 912) and 5.5 (*n* = 14 578) specific peaks. (**C**) iM-IP-qPCR assay for 8 randomly selected pH 5.5 and 7.0 specific iM peaks (P5-1–P5-4 for pH5.5 specific peaks; P7-1–P7-4 for pH 7.0 specific peaks relative to the same negative control (N1) under pH 5.5 and 7.0, respectively. Fold change (P/N1) indicating the enrichment levels of the given positive locus relative to N1. Significance test was determined by using one-way ANOVA analysis, *** *P* < 0.001, ** *P* < 0.01, * *P* < 0.05, ns: no significance. (**D**) iMab antibody-based dot blotting assays for pH 5.5 specific (left, C5-1–C5-5) and pH 7.0 specific (right, C7-1–C7-5) iM peaks. Significance test was determined by using one-way ANOVA analysis, *** *P* < 0.001, ** *P* < 0.01, * *P* < 0.05, ns: no significance. (**E**) CD spectrum assay of two pH 5.5 or 7.0 specific iM peaks at two different pH conditions.

iMs have been detected in human cells under normal cellular environment by using iMab antibody based immunodetection (48). A global profiling of iMs has been reported under physiological conditions (pH 7.4 or 7.5) in humans (iM-ChIP-seq and CUT&TAG) (40,49), and under acidic conditions (pH 5.5, iM-IP-seq) in rice (32). To profile rice iMs at pH 7.0, iM-IP-seq was conducted for identification of *in vitro* iMs reconstituted at pH 7.0. Two well-correlated (*r* = 0.89) biological replicates of iM-IP-seq data (Supplementary Figure S1) were generated and merged to maximize iM peak calling. We obtained 57676 and 94627 *in vitro* iM peaks relative to input (the total DNA before IP) and IgG (the negative non-specific binding control) as controls, and 55 062 common peaks (Figure 1A, left). For in parallel comparisons, we reanalyzed our previous pH 5.5 related iM-IP-seq data (GSE184783) (32), and obtained 33455 common peaks (Figure 1A, right). To better exploit these new data sets, all *in vitro* iMs were divided into three subtypes, including 18 877/19 150 common peaks corresponding to 199 579/150 736 PiMFSs for pH 5.5 and 7.0, respectively (Figure 1B, Supplementary Table S1), 33 455 and 55 062 pH 5.5 and 7.0 specific peaks corresponding to 290 015 and 326 373 PiMFSs, respectively (Supplementary Table S1). A representative Integrative Genomics Viewer (IGV) spanning a 61 kb region from the rice Chr. 1 illustrates the reproducibility of each subtype of iMs between two replicates at two pH conditions (Supplementary Figure S2).

To validate the detection accuracy and exclude the possibility of biased iMs caused by different IP efficiency, 4 of each pH-specific iM peaks were selected for qPCR validation using iM-IPed DNA at either pH 5.5 or 7.0, respectively. By using the same negative control (N1 or N2) under both pH conditions, we observed that pH 7.0 iM peaks were more enriched at pH 7.0 than the corresponding ones at pH 5.5 and *vice versa* (Figure 1C, Supplementary Figure S3). As compared to the negative control, only one pH 7.0 specific iM peaks (P7-1) were not enriched at pH 5.5 (Figure 1C, right) and inconsistency of P5-2 iM between biological replicates relative to N2 at pH 5.5 (Supplementary Figure S3), the remaining ones were enriched at both pH conditions (Figure 1C, Supplementary Figure S3). We then synthesized 8 DNA oligonucleotides corresponding to pH 5.5 and 7.0 specific iM peaks for dot blotting assays; all pH 5.5 or 7.0 specific iMs exhibited stronger signals as compared to the counterpart at pH 7.0 or 5.5 (Figure 1D). Furthermore, we conducted CD (circular dichroism) spectroscopy assays using two synthetic DNA oligonucleotides corresponding to pH 5.5/7.0 specific iM peaks, respectively. pH 5.5 specific iMs exhibited higher ellipticity at *ca*. 290 nm than the ones reconstituted at pH 7.0, while 7.0 pH specific iMs showed a shifted peak with the highest ellipticity at *ca*. 275 nm (Figure 1E). These results indicate that a subset of iMs are formed in a pH dependent manner.

### Distinct genomic features between pH 5.5 and 7.0 biased i-motifs

**Figure 2.**
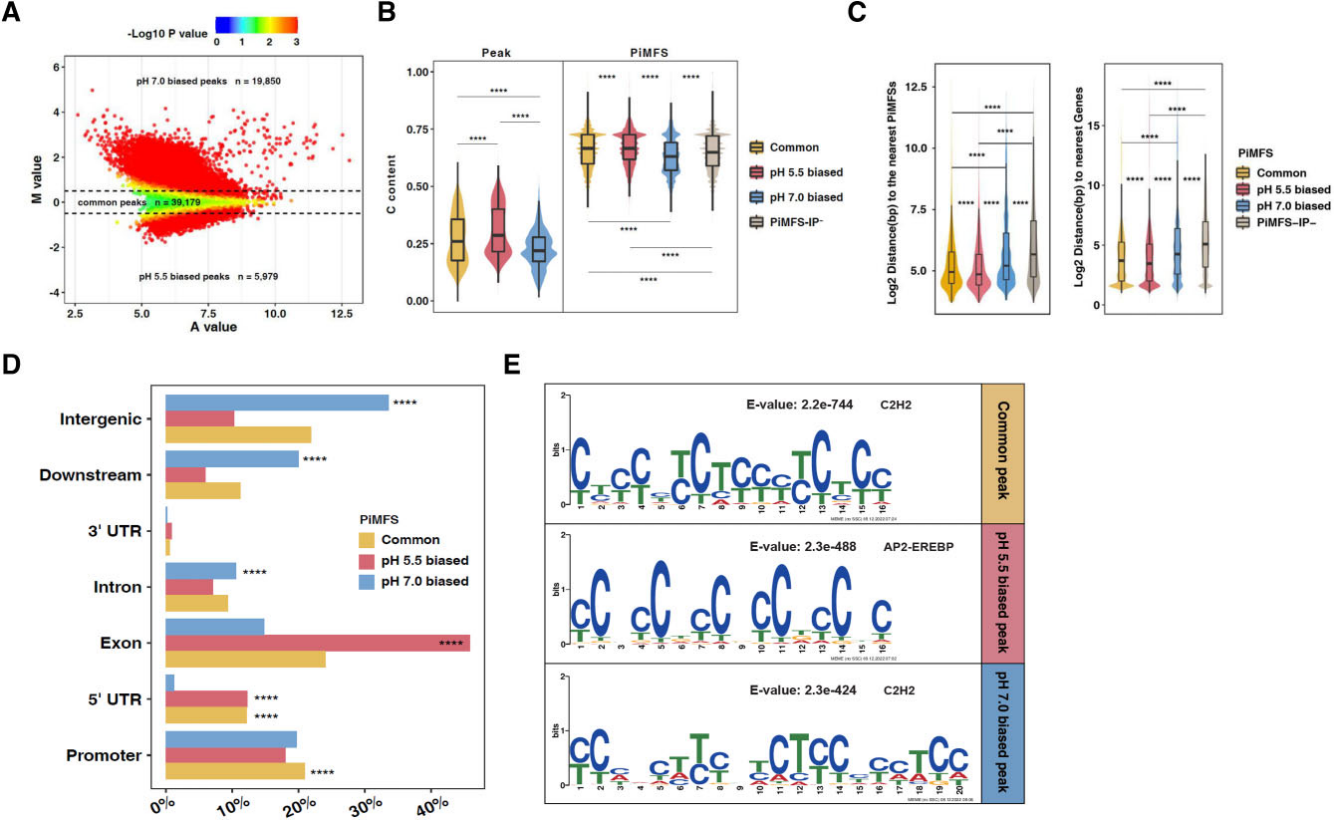
Characterization of genomic features associated with common, pH 5.5 and 7.0 biased iM peaks or PiMFSs-IP^+^/IP^−^(control). (**A**) MA plots for identification of pH 5.5 (*n* = 59 79) and 7.0 biased (*n* = 19 850) and unbiased (common) iM peaks (*n* = 39 179). (**B**) C content of common (yellow), pH 5.5 (red) and 7.0 (blue) biased iM peaks, and the corresponding PiMFSs. **** between the two adjacent violin groups (the left panel) indicating significant difference between the two groups next to each other. (**C**) Distance of common (yellow), pH 5.5 (red) and 7.0 (blue) biased PiMFSs-IP^+^ and PiMSs-IP^−^ (grey, left) to the nearest PiMFSs (left) or genes (right). The distance represents the distance from the center of one PiMFS to the center of the nearest PiMFS within iM peaks and genes for each group. **** between the two adjacent violin groups indicating significant difference between the two groups next to each other. (**D**) Enrichment levels of common (yellow), pH 5.5 (red) and 7.0 (blue) biased PiMFSs-IP^+^ in each subgenomic region as indicated. (**E**) *De novo* motif identification from common (yellow), pH 5.5 (red) and 7.0 (blue) biased iM peaks. Significance test in B–D was determined by using Wilcoxon rank-sum test. ** *P* < 0.01, **** *P* < 0.0001.

It has been documented that intrinsic DNA sequences affect iM stability at different pH conditions (72). However, such investigations remain to be demonstrated on a genome-wide scale. To address this, according to the MA plot we divided all *in vitro* iMs into three subtypes, 5979 and 19 850 for pH 5.5 and 7.0 biased iMs, corresponding to 55 778 and 72 649 PiMFSs, respectively and 39 179 common ones (Figure 2A, Supplementary Table S2). We then calculated the peak/PiMFS length, C content, distance to the nearest PiMFSs/genes for each subtype of iMs. pH 7.0 biased iMs had the lowest C content (Figure 2B), and the longest length of iM peaks and PiMFSs (Supplementary Figure S4A, S4B); they had the second longest distance to the nearest PiMFSs, but the shortest distance to the nearest genes (Figure 2C). In contrast, pH 5.5 biased iMs had the highest C content (Figure 2B) and the shortest distance to the nearest genes (Figure 2C, right). Also, distinct GC content and GC/AT skew were noted between pH 5.5 and 7.0 biased PiMFSs, with the lowest and highest GC content for pH 7.0 and pH 5.5 biased PiMFSs, respectively (Supplementary Figure S4C).

To examine genomic distributions of each subtype of iMs, we calculated the percentage of PiMFSs-IP^+^, representing PiMFSs pulled down with the iMab antibody, distributed in each indicated subgenomic region. As compared with all regular PiMFSs (Supplementary Figure S5), pH 5.5 biased PiMFSs-IP^+^ were more distributed in exons and 5′UTRs, while pH 7.0 biased PiMFSs-IP^+^ were more enriched in introns, intergenic and downstream regions (Figure 2D). Moreover, all regular PiMFSs tended to be distributed more at exons, promoters, intergenic and downstream regions (Supplementary Figure S5). It has been reported that iMs can potentially act as *cis*-regulatory elements (CREs) in the regulation of gene expression in both humans and rice (73,74). To assess potential roles of each subtype of iMs in the regulation of gene transcription, we conduced *de novo* motif identification, and detected the CTCC motif for binding of C2H2 TFs and CCCC for binding of AP2-EREBP TFs were significantly enriched in pH 7.0 biased/common and 5.5 biased iMs, respectively (Figure 2E). These results show that iMs folded at either pH 7.0 or pH 5.5 have distinct genomic features, which may correspond to various biological implications, notably through differential TF bindings.

### Distinct DNA methylation between pH 5.5 and 7.0 biased i-motifs

**Figure 3.**
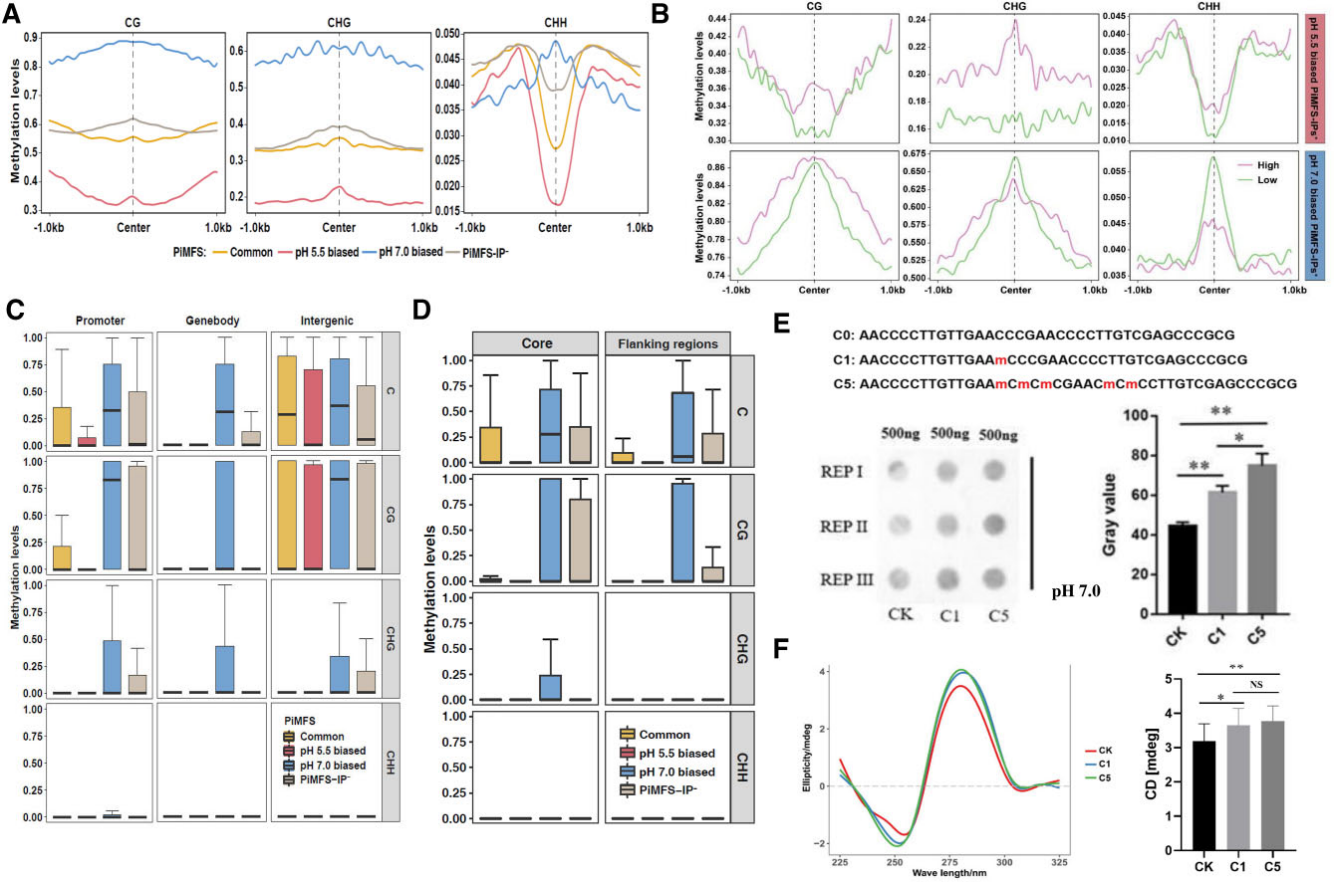
DNA methylation levels of common, pH 5.5 and 7.0 biased PiMFSs-IP^+^ and PiMSs-IP^−^. (**A**) CG, CHG, and CHH methylation levels around ±1 kb of the center of common (yellow), pH 5.5 (red) and 7.0 (blue) biased PiMFSs-IP^+^ and PiMSs-IP^−^ (grey, control). (**B**) CG, CHG, and CHH methylation levels were calculated around ±1 kb of the pH 5.5 and 7.0 biased PiMFSs with high and low read density of iM-IP-seq. (**C**) C, CG, CHG and CHH methylation levels of common (yellow), pH 5.5 (red) and 7.0 (blue) biased PiMFSs-IP^+^ and PiMSs-IP^−^ (grey) distributed in promoters, gene bodies and intergenic regions. (**D**) C, CG, CHG and CHH methylation levels for the core and flanking regions of common (yellow), pH 5.5 (red) and 7.0 (blue) biased PiMFSs-IP^+^ and PiMSs-IP^−^ (grey, control). (**E**) iMab based dot blotting detection of synthesized oligonucleotides containing one 5mC (C1), five 5mCs (C5) sites or unmethylated Cs (CK) at pH 5.5 and 7.0 conditions. (**F**) CD spectrum assay using synthesized oligonucleotides as we did for dot blotting assays in (E) at pH 5.5 and 7.0 conditions. Significance test was determined by using one-way ANOVA analysis, *** *P* < 0.001, ** *P* < 0.01, * *P* < 0.05, ns: no significance.

It has been reported that DNA 5mC exhibits intricate impacts on the stability of iMs in humans and plants, depending on both its genomic position and numbers (32,50,75). Given that pH 5.5 and 7.0 biased iMs had contrasted C content, we explored DNA methylation differences between pH 5.5 and 7.0 biased iMs by calculating DNA methylation levels in each cytosine context (CG, CHG and CHH, where H = A, C and T) across ± 1 kb of the center of each subtype of pH biased PiMFSs. We found that pH 7.0 biased PiMFSs had the highest CG and CHG methylation levels across all regions examined, and had the highest CHH methylation levels around the center (Figure 3A). We did similar analyses for methylation levels of each cytosine context only within PiMFS regions, and obtained a similar trend as these in Figure 3A (Supplementary Figure S6). We then divided each biased iMs with similar C content (Supplementary Figure S7) into 2 subclasses according to the peak abundance (high and low) for conducting similar analyses as we did in Figure 3A. Strikingly, we found that pH 5.5 biased PiMFSs exhibited a positive association with methylation levels within each cytosine context (Figure 3B, top), while pH 7.0 biased PiMFSs were positively associated with CG methylation levels, but negatively associated with CHH methylation levels. Moreover, we also found a negative association of CHG methylation at ± 250 bp of the center, and a positive association at the flanking regions (Figure 3B, bottom). Similar to pH 5.5 biased PiMFSs, common PiMFSs exhibited a positive association with CG, CHG and CHH methylation levels even though CHH methylation levels in the center were much lower as compared to the flanking regions (Supplementary Figure S8). These results suggest differential impacts of DNA methylation on the stability or formation of pH biased iMs.

Furthermore, we counted DNA methylation levels in each C context for each subtype of iMs distributed in promoters, gene bodies and intergenic regions. pH 7.0 biased iMs displayed the highest DNA methylation levels in all C contexts in promoters and gene bodies, while pH 5.5 biased iMs had the lowest DNA methylation levels in promoters and gene bodies (Figure 3C). Moreover, a similar trend was observed among subtypes of PiMFSs with similar total C, CG, CHG and CHH content, respectively (Supplementary Figure S9). These results indicated that differences in DNA methylation levels between pH 5.5 and 7.0 biased iMs were genomic region dependent. We conducted dot blotting assays using the same amount of genomic DNA reconstituted at either pH 5.5 or 7.0, and observed stronger (increased by 18%) immuno-signals in pH 7.0 than pH 5.5 conditions (Supplementary Figure S10), which is in line with higher numbers of peaks identified at pH 7.0 as compared to pH 5.5 (Figure 1B). These observations are critical since they tend to show that pH 7.0 favors a subset of iM formation as compared to pH 5.5, which is rather counter-intuitive with regard to the wealth of data collected about iM formation *in vitro*.

To assess impacts of DNA methylation position on iM formation, we calculated CG, CHG and CHH methylation levels within the core and flanking regions of each subtype of PiMFSs (Supplementary Figure S11). The core regions of pH 7.0 biased PiFMSs had the highest C, CG and CHG methylation levels, and their flanking regions had the highest C and CG methylation levels among subtypes of iMs examined. In contrast, pH 5.5 biased PiMFSs had much lower C, CG and CHG methylation levels (Figure 3D). After a closer examination of these results, we found that DNA methylation levels tended to be higher in the core regions than the side regions for each subtype of iMs, especially for pH 7.0 biased PiMFSs (Figure 3D). To further confirm the impact of DNA methylation in the core regions on iM formation at pH 7.0, we conducted dot blotting assays using synthetic DNA oligonucleotides with one (C1) and five (C5) methylated C folded at pH 7.0. We found that the intensity of immuno-signal exhibited a direct association with C methylation levels (Figure 3E). Similarly, CD spectroscopy assays showed that DNA methylation levels tended to be directly associated with the amplitude of CD signal at *ca*. 290 nm among oligoes with different DNA methylation levels even though only a subtle difference occurred between C1 and C5 oligoes (Figure 3F). These results thus contrasted with the overall negative associations between DNA methylation and iM formation at pH 5.5 (32). Furthermore, we observed that iMs with medium methylation levels for pH 5.5 biased iMs and low methylation levels for pH 7.0 biased iMs had the highest normalized read density (Supplementary Figure S12A); and pH 7.0 biased iMs with low DNA methylation levels had much higher CG and CHG methylation levels than pH 5.5 biased ones (Supplementary Figure S12B). These results suggest that only a certain range of DNA methylation levels may facilitate iM formation under pH 5.5 or 7.0.

To examine the binding affinity of iMab to iMs formed with methylated and unmethylated under pH 5.5 and 7.0, respectively, we first conducted CD assays to obtain two oligoes (unmethylated CK, and M1 with one methylated C) with similar potentials of iM formation between pH 5.5 and 7.0 for iMab antibody-based dot blotting assays (Supplementary Figure S13A). We found that two oligoes reconstructed under pH 5.5 and 7.0 exhibited similar immuno signals under genomic IP buffer with pH 5.5 or pH 7.0 conditions (Supplementary Figure S13B). These results indicated a similar binding affinity of iMab antibody to oligoes unmethylated or with one C methylated between pH 5.5 and 7.0.

Taken together, these results show that pH 7.0 biased iMs have overall higher DNA methylation levels as compared to those formed at pH 5.5. DNA methylation levels differently influence iM formation at both pH conditions, indicating that DNA methylation may be a key factor for controlling iM formation under physiological conditions.

### Impacts of DNA demethylation on i-motif formation at pH 5.5 and 7.0 conditions

**Figure 4.**
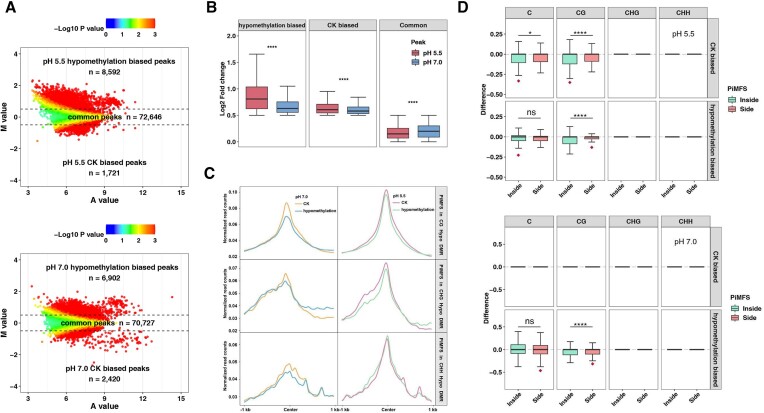
Impacts of DNA hypomethylation on iM formation at pH 5.5 and 7.0 conditions. (**A**) MA plots for identification of hypomethylation biased iM peaks at pH 5.5 and 7.0 conditions, respectively, including 8529/1721 hypomethylation and CK biased iM peaks at pH 5.5 conditions, respectively; 6902/2420 hypomethylation/CK biased iM peaks at pH 7.0 conditions, respectively. (**B**) Fold changes of normalized iM-IP-seq read density of hypomethylation over CK biased and common peaks at pH 5.5 (red) or 7.0 (blue) conditions. Significance test was determined by using Wilcoxon rank-sum test. **** *P* < 0.0001. (**C**) Normalized read counts of iM-IP-seq for CK and hypomethylation were plotted around ±1 kb of the center of PiMFSs associated with CG, CHG or CHH hypo DMRs at pH 5.5 and 7.0 conditions. (**D**) C, CG, CHG and CHH methylation changes of CK and hypomethylation biased PiMFSs within core (green) and flanking regions (red) of iMs at pH 5.5 (upper) and 7.0 (down) conditions. Significance test was determined by using Wilcoxon rank-sum test. * *P* < 0.05, **** *P* < 0.0001, ns: no significance. The red diamonds under the box indicating the group with a larger average difference value.

To investigate the impact of DNA demethylation on iM formation, we conducted dot blotting assays using demethylated genomic DNA from zebularine treatment, a DNA methyltransferase inhibitor (56). The signal intensity of demethylated DNA was increased by *ca*. 30% and 36% at pH 5.5 and 7.0, respectively, as compared to the corresponding CK (Supplementary Figure S14). This suggests that a global DNA demethylation tends to promote iM formation genome-wide. To further investigate this, we conducted iM-IP-seq using CK and demethylated DNA, which led to two well-correlated (*r* = 0.93 and 0.98) biological data sets at pH 5.5 and 7.0 (Supplementary Figure S15, Supplementary Table S3). According to MA plotting using merged data, we obtained 72 646 and 70 727 common peaks for pH 5.5 and 7.0, respectively, and obtained 1721 and 2420 CK biased peaks, corresponding to 17 646 and 15 678 PiMFSs, and 8592 and 6902 demethylation biased peaks, corresponding to 21 150 and 45 289 PiMFSs, at pH 5.5 and 7.0, respectively (Figure 4A, Supplementary Table S3). Demethylation and CK biased peaks exhibited higher normalized read intensity than the corresponding loci in CK and demethylated DNA at both pH conditions, and almost no significant change occurred in common peaks between CK and demethylated DNA at pH 5.5, while common peaks in CK exhibited higher read intensity than these from demethylated DNA at pH 7.0 (Supplementary Figure S16). We calculated fold change of normalized iM-IP-seq read density of demethylation and CK biased peaks, and common peaks, and found that demethylation and CK biased peaks exhibited higher fold changes of the normalized read density of hypomethylation over CK biased peaks at pH 5.5 than these at pH 7.0, and an opposite trend occurred for common peaks at both pH conditions (Figure 4B).

We further analyzed newly generated data of bisulfite sequencing (BS-seq) from CK and demethylated DNA, and obtained 182 125 CG, 3868 CHG and 3890 CHH hypo-DMRs (differentially methylated regions), respectively (Supplementary Table S4). After plotting the normalized iM-IP-seq read counts across ±1 kb of the center of the PiMFSs containing hypo CG/CHG/CHH DMRs at both pH conditions, we found that DNA demethylation exhibited a decreased occupancy of normalized reads as compared to CK at pH 5.5 and 7.0 (Figure 4C). Differences of read occupancy between CK and demethylated DNA were higher at pH 7.0 than pH 5.5. To assess impacts of position related DNA demethylation changes on iM formation, we calculated differences of DNA methylation levels for the core and flanking regions of biased PiMFSs at both pH conditions. We observed almost no change in CHG and CHH methylation levels for the core and flanking regions of all biased PiMFSs at both pH conditions, and the total C and CG methylation levels for CK biased ones at pH 7.0 (Figure 4D); the average value of CG methylation levels in the core regions exhibited higher differences for CK biased ones at pH 5.5, while had less differences for demethylation biased ones in both pH conditions as compared to the flanking regions of the corresponding PiMFSs (Figure 4D).

Taken together, these results indicate that DNA demethylation had more impacts on a subset of iM formation at pH 7.0 than pH 5.5. The core regions tend to have more CG methylation changes than the flanking regions for CK biased PiMFSs at pH 5.5, but an opposing trend for demethylation biased ones at both pH conditions.

### Impacts of DNA hypermethylation on i-motif formation at pH 5.5 and 7.0 conditions

**Figure 5.**
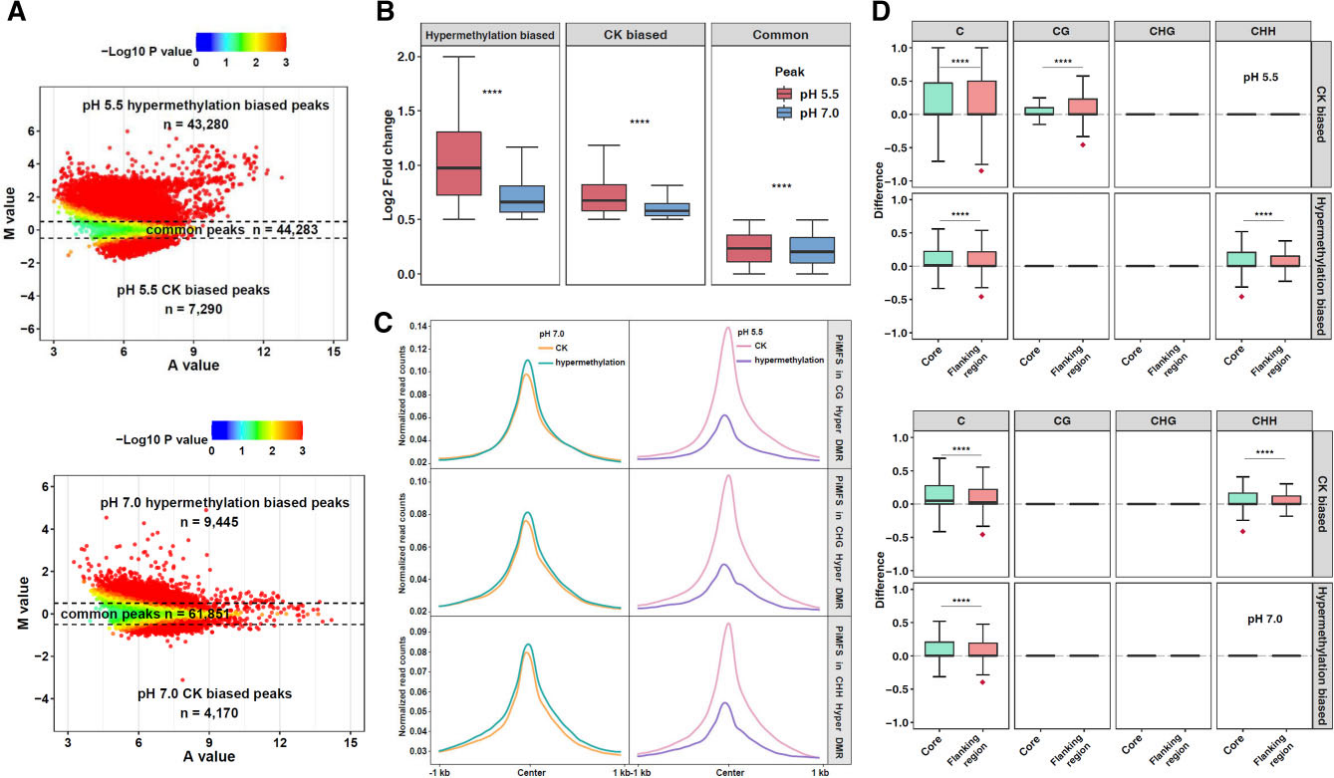
Impacts of DNA hypermethylation on i-motif formation at pH 5.5 and 7.0 conditions. (**A**) MA plots for identification of hypermethylation biased iM peaks at pH 5.5 and 7.0 conditions, including 432 80/7290 hypermethylation and CK biased iM peaks at pH 5.5 conditions, respectively; 9445/4170 hypermethylation/CK biased iM peaks at pH 7.0 conditions, respectively. (**B**) Fold changes of normalized iM-IP-seq read density of hypermethylation/CK biased and common peaks at pH 5.5 (red) or 7.0 (blue) conditions. Significance test was determined by using Wilcoxon rank-sum test. **** *P* < 0.0001. (**C**) Normalized read counts of iM-IP-seq for CK and hypermethylation were plotted around ±1 kb of the center of PiMFSs associated with CG, CHG and CHH hyper DMRs at pH 5.5 and 7.0 conditions. (**D**) C, CG, CHG and CHH methylation changes of CK and hypermethylation biased PiMFSs within the core (green) and flanking regions (red) of iMs at pH5.5 (upper) and 7.0 (down) conditions. Significance test was determined by using Wilcoxon rank-sum test. **** *P* < 0.0001. The red square under the box indicating the group with a larger average difference value.

To assess how DNA hypermethylation affects iM formation, we used GpC methyltransferase M.Cvip I enzyme (76), which seems to also methylate CHG and CHH context in the plant genome (Supplementary Figure S17), to treat genomic DNA, resulting in a global increase of DNA methylation levels through dot blotting assays (Supplementary Figure S18A), while a decrease of iM dot immunosignal from M.Cvip I treated DNA by 28% at pH 5.5 and 17% at pH 7.0 as compared to CK (Supplementary Figure S18B). We again generated two well-correlated (*r* = 0.94 and *r* = 0.98) biological iM-IP-seq data using M.Cvip I treated DNA at pH 5.5 and 7.0, respectively (Supplementary Figure S19). According to MA plotting using merged data, we obtained 44 283 and 61 851 common peaks, 7290 and 4170 CK biased peaks, corresponding to 95 847 and 21 937 PiMFSs, and 43280 and 9445 hypermethylation biased peaks, corresponding to 117 176 and 146 572 PiMFSs, relative to input and IgG as controls at pH 5.5 and 7.0, respectively (Figure 5A, Supplementary Table S3). As expected, hypermethylation and CK biased peaks exhibited higher normalized read intensity than the corresponding CK and demethylation related loci at both pH conditions, while almost no significant change occurred in common peaks between CK and hypermethylation at pH 7.0 conditions, and a higher read intensity was obtained for common CK peaks as compared to those under hypermethylation conditions at pH 5.5 (Supplementary Figure S20). After calculating fold changes of normalized iM-IP-seq read density of hypermethylation/CK biased and common peaks, all peaks exhibited higher fold changes at pH 5.5 than at pH 7.0 (Figure 5B).

After analyzing newly generated BS-seq data from CK and hypermethylated DNA, we obtained 115671 CG, 283720 CHG and 656623 CHH hyper-DMRs, respectively (Supplementary Table S4). We then plotted normalized iM-IP-seq read counts at ± 1 kb of the center of the PiMFSs containing hyper CG/CHG/CHH DMRs at both pH conditions, and found that PiMFSs associated with hyper-DMRs overall exhibited an increased read occupancy as compared to CK at 7.0, in sharp contrast with what happened at pH 5.5 (Figure 5C). We also found that differences of read occupancy between CK and hypermethylated DNA were higher at pH 5.5 than 7.0. To assess impacts of position related DNA hypermethylation changes on iM formation, we calculated differences in DNA methylation levels for the core and flanking regions of biased PiMFSs at both pH conditions. We found that the flanking regions of all biased PiMFSs in both pH conditions had a higher average difference value for total methylated C levels than the corresponding core regions; the average difference value of CG methylation levels in CK biased one at pH 5.5 was higher in the flanking regions than the core regions, while the core regions displayed a higher average difference value of CHH methylation levels in hypermethylation biased PiMFSs at pH 5.5 and CK biased ones at pH 7.0 than the corresponding ones in the flanking regions (Figure 5D). The remaining comparisons were almost no methylation changes at pH 5.5 and/or 7.0 conditions (Figure 5D). In addition, we assessed impacts of hyper or hypomethylation on pH 5.5 (*n* = 5979) or 7.0 (*n* = 19 850) biased iMs identified in Figure 2A under the same pH conditions, respectively (Supplementary Figure S21A), and found that hypermethylation resulted in loss of approximately 60% of pH 5.5 biased iMs, and hypomethylation resulted in loss of approximately 3% and gain of approximately 16% of pH 7.0 biased iMs (Supplementary Figure S21B). Hypermethylation related methylation changes of CG, CHG and CHH were higher in loss of pH 5.5 biased iMs as compared to retained iMs under pH 5.5 conditions (Supplementary Figure S21C, left), by contrast, hypomethylation related methylation changes of CG and CHG were less in loss of pH 7.0 biased iMs as compared to retained and gained iMs under pH 7.0 conditions (Supplementary Figure S21C right). After looking into methylation levels of CG, CHG and CHH for hypomethylation related loss, retain and gain of pH 7.0 biased iMs under pH 7.0 conditions, we found that hypomethylation reduced CG methylation levels in loss of iMs, and CG/CHG methylation levels in retain and gain of iMs; and CG and CHG methylation levels for loss of iMs in CK or hypomethylation were less than the corresponding retain and gain of iMs (Supplementary Figure S21D). These analyses further suggest that only a certain range of DNA methylation levels may facilitate iM formation under pH 5.5 or 7.0, thus PiMSFs with DNA methylation levels out of the range will reduce folding potentials, leading to dynamics of the corresponding iMs with hyper or hypomethylation under pH 5.5 or 7.0.

Collectively, these results indicate that DNA hypermethylation has distinct impacts on iM formation between pH 5.5 and 7.0, being more influential at pH 5.5 than 7.0. The core regions of CK biased PiMFSs tend to have less CG methylation changes at pH 5.5, but have more CHH methylation changes for hypermethylation biased ones at both pH conditions as compared to the flanking regions of the corresponding PiMFSs.

### pH dependent impacts of DNA hyper/hypo-methylation on i-motif formation and pH dependent transcriptomic reprogramming

**Figure 6.**
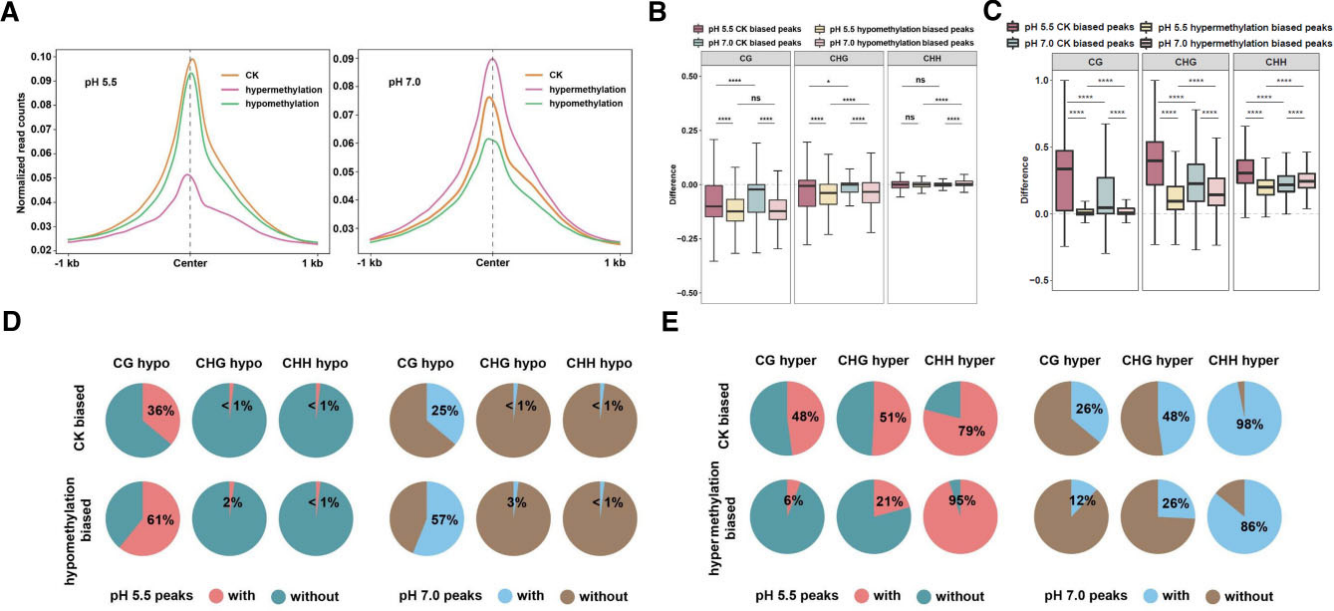
Characterization of pH dependent impacts of DNA hyper/hypo-methylation on iM formation. (**A**) Normalized read counts of iM-IP-seq for CK (orange), hypermethylation (pink) and hypomethylation (green) at pH 5.5 (left) and 7.0 pH (right) conditions were plotted around ±1 kb of the center of all PiMFSs. (**B**) Differences of CG, CHG and CHH methylation levels among pH dependent CK and hypomethylation biased peaks, including pH 5.5 biased CK peaks (red), pH5.5 biased hypomethylation peaks (yellow), pH 7.0 biased CK peaks (blue) and pH 7.0 biased hypomethylation peaks (pink). (**C**) Differences of CG, CHG and CHH methylation levels among pH dependent CK and hyper-methylation biased peaks, including pH 5.5 biased CK peaks (red), pH 5.5 biased hyper-methylation peaks (yellow), pH 7.0 biased CK peaks (blue) and pH 7.0 biased hyper-methylation peaks (pink). Difference = 5mC levels with hypomethylation/hypermethylation—5mC levels in CK. (**D**) Venn plots illustrating distributions of CG/CHG/CHH hypo-DMRs between CK and hypomethylation biased peaks at pH 5.5 (left) and pH 7.0 (right) conditions. (**E**) Venn plots illustrating distributions of CG/CHG/CHH hyper-DMRs between CK and hyper-methylation biased peaks at pH 5.5 (left) and pH 7.0 (right) conditions. Significance test in (B) and (C) was determined by using Wilcoxon rank-sum test. * *P* < 0.05, **** *P* < 0.0001, ns, no significance.

To go a step further, we plotted the normalized read counts of hyper or hypomethylated and CK DNA at ±1 kb of the center of all regular PiMFSs. We found that the intensity of hypermethylated DNA was the lowest at pH 5.5, but the highest at pH 7.0, by contrast, the intensity of hypomethylated DNA was the second highest at pH 5.5, but the lowest at pH 7.0 (Figure 6A). Also, CK and demethylation biased peaks tended to have higher DNA methylation levels in each C context at pH 5.5 than the corresponding biased ones at pH 7.0; moreover, demethylation biased peaks tended to have lower DNA methylation levels in each cytosine context as compared to the corresponding CK biased ones at pH 5.5 or 7.0 (Supplementary Figure S22). In contrast, hypermethylation biased peaks tended to have higher DNA methylation levels in each cytosine context as compared to the corresponding CK biased ones at pH 5.5 or 7.0 conditions (Supplementary Figure S23). To examine impacts of methylation changes on biased iM formation, we calculated methylation changes in each cytosine context by using methylation levels of hyper or hypomethylated DNA minus CK DNA as indicated. We found that demethylation biased peaks at pH 5.5 or 7.0 exhibited higher CG and CHG methylation changes as compared to CK biased ones (Figure 6B). In contrast, pH 5.5 hypermethylation biased peaks at pH 5.5 or 7.0 exhibited less CG and CHG methylation changes as compared to the corresponding CK biased ones; CK biased peaks at pH 5.5 had the highest methylation changes in each cytosine context (Figure 6C). Furthermore, we assessed impacts of hyper or hypomethylation on pH dependent iM dynamics as shown in Supplementary Figure S24A, and found that hypermethylation resulted in loss of approximately 19% of pH 5.5 biased iMs under pH 7.0 conditions, and hypomethylation resulted in loss of approximately 38% of pH 7.0 biased iMs under pH 5.5 conditions (Supplementary Figure S24B). We then examined hyper- or hypo-methylation related DNA methylation changes among loss and retain of iMs as shown in Supplementary Figure S4C. We found that hypermethylation related methylation changes of CG, CHG and CHH were less in loss of pH 5.5 biased iMs as compared to retained ones under pH 7.0 conditions (Supplementary Figure S24C top), similarly, hypomethylation related loss of pH 7.0 biased iMs conditions exhibited less methylation changes of CG and CHG as compared to these retained ones under pH 5.5 (Supplementary Figure S24C bottom).

To examine impacts of cytosine context related methylation changes on biased iM formation, we calculated overlapping ratios of biased peaks with CG/CHG/CHH hypo- or hyper-DMRs at both pH conditions, respectively. We found that 36%/61%, and 25%/47% CG hypo-DMRs were overlapped with CK and demethylation biased peaks, respectively, at pH 5.5 and 7.0 (Figure 6D). In contrast, CK or hypermethylation biased peaks were overlapped CG/CHG/CHH hyper-DMRs at both pH conditions, with a descending order from CHH, CHG to CG hyper-DMRs (Figure 6E). Moreover, hypermethylation biased peaks had a lower percentage of CG/CHG hyper-DMRs at both pH conditions and CHH hyper-DMRs at pH 7.0, but a higher percentage of CHH hyper-DMRs at pH 7.0 as compared to the corresponding CK biased peaks (Figure 6E).

It has been proposed that pH may act as a signal or secondary messenger involved in the regulation of plant growth and development (77). To assess pH dependent impacts on plant growth, we examined phenotypic changes of rice seedlings grown under pH 5.5 and 7.0, and found that seedlings under pH 7.0 exhibited overall shorter plant height but slightly longer primary roots with a little darker color as compared to these under pH 5.5. To investigate pH dependent transcriptomic reprogramming, we conducted biologically replicated RNA-seq data sets from rice seedlings (Supplementary Figure S25A), leading to 697 and 414 genes highly expressed under pH 5.5 and 7.0, respectively (Supplementary Figure S25B). According to GO term enrichment analyses, DEGs under pH 5.5 had more enriched GO terms related to responses to oxidative stress and chromatin assembly etc., by contrast, DEGs under pH 7.0 were more enriched in GO terms associated with oxidation reduction and cellular nitrogen etc. (Supplementary Figure S25C). To assess if iMs are involved in the regulation of pH related DEGs, we analyzed PiMFSs from DEGs and found that DEGs under pH 7.0 had slightly more PiMFSs in promoters only (4.8% vs. 3.2%), but less in gene bodies & promoters (72% versus 77%), and gene bodies only (20% versus 23%) as compared to these under pH 5.5 (Supplementary Figure S25D). DGEs under pH 5.5 exhibited a higher density of PiMFSs than these under pH 7.0 (Supplementary Figure S25E). Furthermore, to examine impacts of pH inducible iMs on pH related differential gene expression, we conducted iMab antibody-based CUT&TAG followed by qPCR assays for 5 DEGs, including *OsNRT1.1b*, *OSA1*, *OSA2*, *OsCML21* and *OsNRT2.4*. 4 of them exhibited a similar trend with iM-IP-seq between pH 5.5 and 7.0 (Supplementary Figure S26A). Importantly, we found that an *in vivo* iM in *OsNRT1.1b*, *OSA1* and *OsCML21* exhibited a direct correspondence with gene expression changes, while an *in vivo* iM in *OSA2* displayed a negative association with gene expression changes between pH 5.5 and 7.0 (Supplementary Figure S26B). These results indicate that a subset of *in vivo* iMs may contribute to pH dependent transcriptomic reprogramming.

Taken together, these results show that DNA demethylation or hypermethylation exhibits pH dependent impacts on iM formation. CG hypo-DMRs and CHH hyper-DMRs alone or coordinated with CG/CHG hyper-DMRs may play determinant roles in the regulation of iM formation at pH 5.5 and 7.0.

## Discussion

This study provided evidence showing that the nature of PiMFSs may play a key role in pH-dependent iM formation (Figure 2A). These sequence features include C and GC content, PiMFSs length and density, and the distance to the nearest PiMFSs or genes. In general, iMs with less C/GC content and longer length but less density of PiMFSs tend to be more stable at pH 7.0 than these preferentially formed at pH 5.5. It has been documented that, in addition to acidic pH driven iM formation, intrinsic DNA sequence features, including loop sequences, C tract length, capping interactions flanking C–C^+^ stack and sequences of minor groove tetrads, may act as determinant factors to stabilize a subset of iMs under neutral or physiological pH conditions (9,10,78–81), such as *HIF-1α* promoter sequences (82), and 17 of biophysically verified human PiMFSs (78). This could be possibly caused by differential impacts of distinct pH conditions on pH induced folding dynamics or structural transitions between duplex and tetrads of iMs with distinct sequence contexts (83,84). For instance, pH 7.0 stabilized iMs contain two C:C^+^ base pairs interacted with two minor groove G:C:G:C tetrads, by contrast, pH 5.5 stabilized ones have longer iM structures containing four C:C^+^ base pairs interacted with two minor groove G:T:G:T tetrads (84,85). Shorter iM structures with additional G:C interactions have more H-bonds as compared to longer iM structures with G:T, thus the former can be stabilized at pH 7.0, but is unstable at pH 5.5 due to presence of more protonated Cs (86). Many external factors have been reported to stabilize iMs under neutral pH conditions, including supercoiling (11), temperatures (16), molecular crowding (5,18), interacting agents (30,31), salt concentration (4,87), and cations such as Ag^+^ (21). Therefore, intrinsic DNA sequences alone or in combination with these external factors determine pH dependent formation of a subset of iMs. Our study also supported recent findings showing that distinct PiMFSs behave differently in response to pH changes (10). We found that iMs that fold specifically at either pH 5.5 or 7.0 exhibited distinct genomic localizations, suggestive of distinct roles in the regulation of gene transcription.

Importantly, our study provided evidence showing how DNA methylation impacts iM formation on a genome-wide scale. It was evidenced by the findings as below: the highest DNA methylation levels for pH 7.0 biased peaks, while the lowest DNA methylation levels for pH 5.5 biased ones; distinct relationships between DNA methylation levels and the intensity of pH 5.5 and 7.0 biased peaks; hyper or demethylation biased iMs exhibited higher fold changes of peak intensity at pH 5.5 than pH 7.0. Moreover, it also provided evidence showing that hyper or demethylation induced a substantial number of biased iMs at pH 5.5 or 7.0 conditions, which is partly affected by distinct changes of CG, CHG or CHH methylation levels alone or combined with pH changes; distinct methylation changes between biased iMs or between the core regions and the flanking regions of biased iMs. For instance, less changes of CG but more changes of CHH methylation levels occurred in the core regions of demethylation and hypermethylation biased iMs, respectively, as compared to the corresponding flanking regions at pH 5.5 and 7.0, while an opposite trend in CG methylation changes occurred for the core regions of CK biased ones relative to the corresponding regions of demethylation and hypermethylation at pH 5.5. These results highlight the intricate influence of the both genetic and epigenetic factors on iM formation, as previously suggested (32).

These results further substantiate previous results demonstrating effects of 5mC on the stabilization of iMs found in the telomere of *Arabidopsis* (34), and that iMs folded at pH 7.0 are generally more methylated than those folded under acidic conditions (33). It is worth noting that our study showed that DNA demethylation can promote substantial numbers of iM formation under both pH conditions (Figure 5A). This seems to be contradictory to previous findings regarding overall promotional roles of 5mC modifications on stabilization of iMs, even though they are formed with specific DNA oligonucleotides such as *VEGF*, *BCL2*, *HRAS1/2*, *c-MYC* and telomeric i-motif structures (5,15,33,53,54). Plausible explanations are as below: 1) 5mC modifications exhibit conditional impacts on iM stability, which are number and position dependent. It has been reported that iMs can be stabilized by a single 5mC modification, while destabilized by hypermethylation (88). Specifically, the human telomeric iM was more stable with a single or paired methylated Cs, but unstable with 2 more 5mCs (15), which can be partly contributed by 5mC reducing but 5hmC enhancing the flexibility of underlying DNA (89). The methyl group of a single or more 5mCs in the major groove restricts DNA bending and twisting due to steric hindrance, thereby affecting the outcome of its shape and TF mediated base readout through conformational changes in the major and minor groove (90–94). Moreover, distinct 5mC positions exhibited completely opposite effects on telomeric iM stabilization in human and *Arabidopsis* (33,34). 2) Demethylation biased iMs under pH 5.5 and 7.0 have higher DNA methylation levels before (Supplementary Figure S27A) and after (Supplementary Figure S27B) C normalization as compared to common iMs in CK, indicating that these sequences are originally hyermethylated, which may create more chances of steric hindrance between paired 5mC-5mC or 5mC-C, thereby leading to inhibition of iM formation under pH 5.5 or 7.0. 3) Unlike the majority of CG context in the mammalian genomes, there are three distinct C contexts (CG, CHG and CHH) in the plant genomes, thus individual or combined methylation of each C context may exert complex impacts on iM formation or stabilization. CG methylation has been reported to induce conformational changes of iMs from CpG islands of four genes related to cancer (*VEGF*, *C-KIT*, *BCL2* and *HRAS*) (54). 4) Unlike already reported studies with limited DNA oligonucleotides, it is still unclear how the intrinsic nature of iM sequences, like loop length and total numbers of Cs, combines with 5mC modifications to affect iM stabilization on a genome-wide scale. It has also been reported that hypomethylation can slightly elevate iM stability (50,54). These results suggest a complex relationship between DNA methylation and iM stability. We postulate here that presence of 5mC could affect the interaction between the C:C^+^ base pair and the minor groove tetrads, in a sequence-dependent manner, which could potentially lead to pH-dependent formation of a subset of iMs. Detailed mechanisms underlying iM stabilization remain to be investigated.

The acid growth theory is a widely accepted viewpoint in the plant community, which is supported by the findings about acidic pH within apoplast (pHapo) facilitates cell expansion, while alkaline pHapo enhances stiffness of cell wall and activates cellular activities against abiotic and biotic stress, leading to inhibition of cell growth (95–97). Ambient pH can alter a plethora of physiological and biochemical processes such as photosynthesis, ionic homeostasis, ROS production, the activities of antioxidant enzymes and reprogramming of gene transcription (98,99), thereby affecting various biological processes in plants, including growth and development, flowering time, nutrition absorption, hormone levels, structures of cell wall, stomatal movement activities, and responses to various biotic and abiotic stresses (95,98,100–103). For instance, acidic conditions promote uptake of micronutrients, while neutral or slightly alkaline pH facilitates absorbance of macronutrients (95,104). Our study showed pH dependent reprogramming of a subset of genes, which is consistent with previous findings (98,105–107). However, it is still unknow the link between pH mediated iM changes and expression of pH responsive genes. Our study provided evidence showing that *in vivo* pH 5.5 and 7.0 preferred iMs may play key roles in the modulation of a subset of pH responsive genes such as *H^+^-ATPase (OSA1/2)* (108), *OsNRT1.1b* (109) and *OsCML21* (110), which are possibly involved in distinct pH related phenotypic changes.

Collectively, our results highlight the different parameters that govern iM formation and stability (sequence, environment, epigenetics) on a genome-wide scale, and the close balance between them that orchestrates pH dependent formation (111–114). We believe that our study will contribute to in-depth understanding of DNA methylation in the modulation of pH dependent dynamics of iM conformations, thus broadening its biological implications and practical applications.

## Supplementary Material

gkad1245_Supplemental_File

## Data Availability

All genomic data produced in this study have been deposited in the NCBI Gene Expression Omnibus (GEO; http://www.ncbi.nlm.nih.gov/geo/) under accession number GSE234752.
