# Supplementary material for: The roles of DNA methylation on pH dependent i-motif (iM) formation in rice
Source: Nucleic Acids Res. 2024 Jan 5;52(3):1243–57. doi: 10.1093/nar/gkad1245 (PMC10853798; doi:10.1093/nar/gkad1245)
Supplement: gkad1245_Supplemental_File [file gkad1245_supplemental_file.pdf]

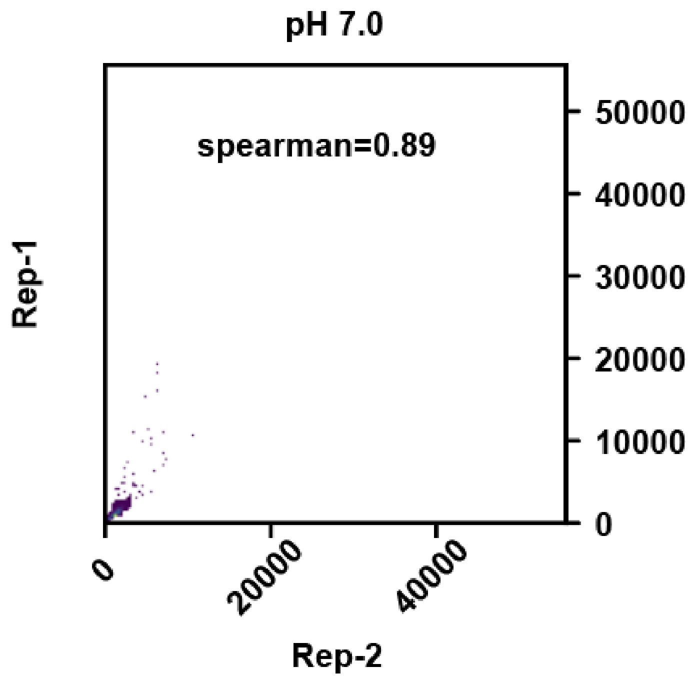

Figure S1 Correlation analyses of two biologically replicated iM-IP-seq data sets at pH 7.0 conditions.

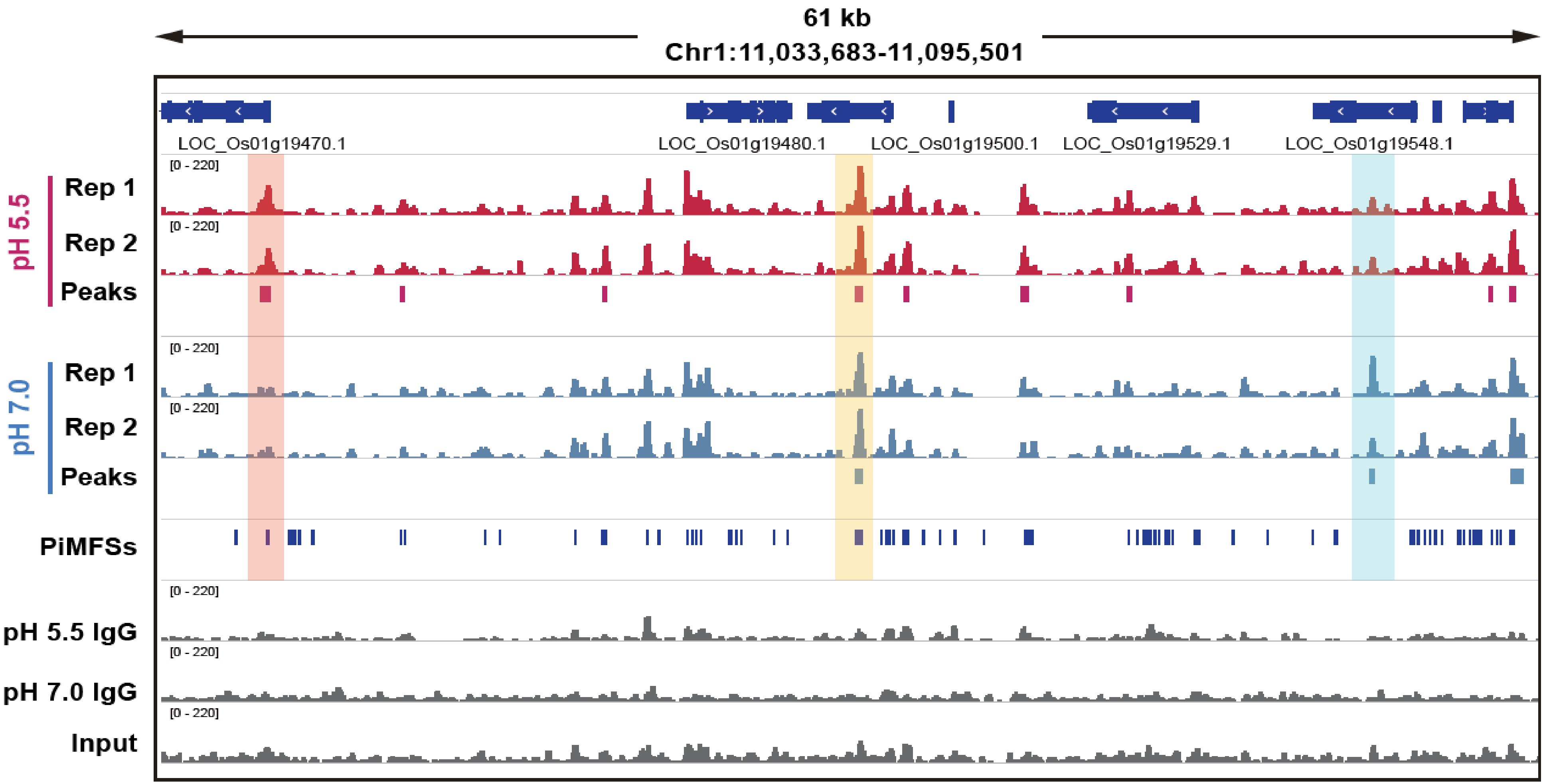

Figure S2 A representative Integrative Genomics Viewer (IGV) snapshot across a 61 kb from the rice Chr. 1 illustrating the reproducibility of iM peaks and PiMFSs at pH 5.5 and 7.0 as indicated. The red, yellow and blue frames indicating the pH 5.5 specific, common and the pH 7.0 specific peaks, respectively.

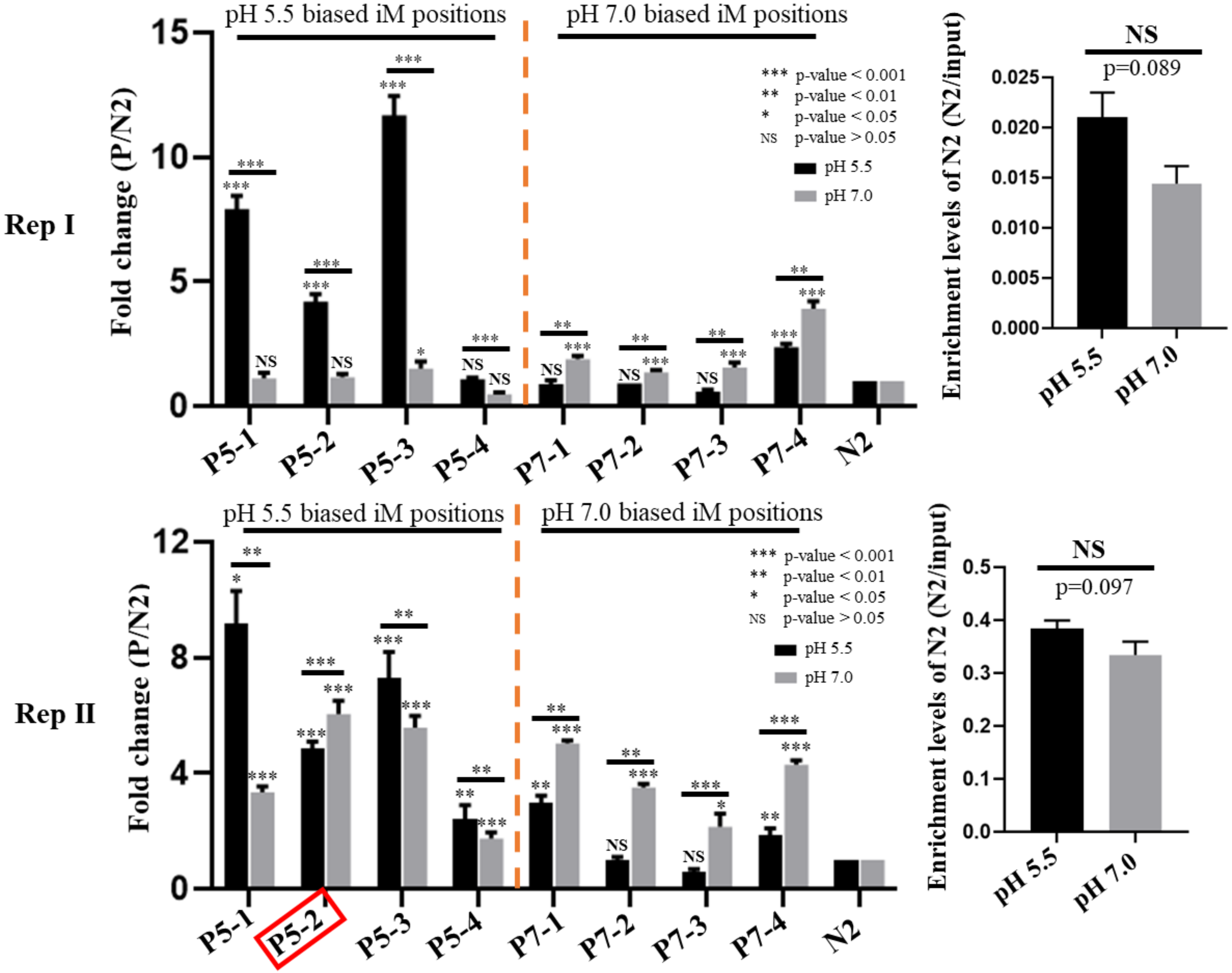

Figure S3 iM-IP-qPCR assay for 8 randomly selected pH 5.5 and 7.0 specific iM peaks (P5-1~P5-4 for pH5.5 specific peaks. P7-1~P7-4 for pH 7.0 specific peaks relative to the same negative control (N2) under pH 5.5 and 7.0, respectively. Fold change (P/N2) indicating the enrichment levels of the given positive locus relative to N2. P5-2 iM marked with a red box was inconsistent between replicates. Significance test was determined by using One-way ANOVA analysis, *** $p < 0.001$, ** $p < 0.01$, * $p < 0.05$, ns: no significance.

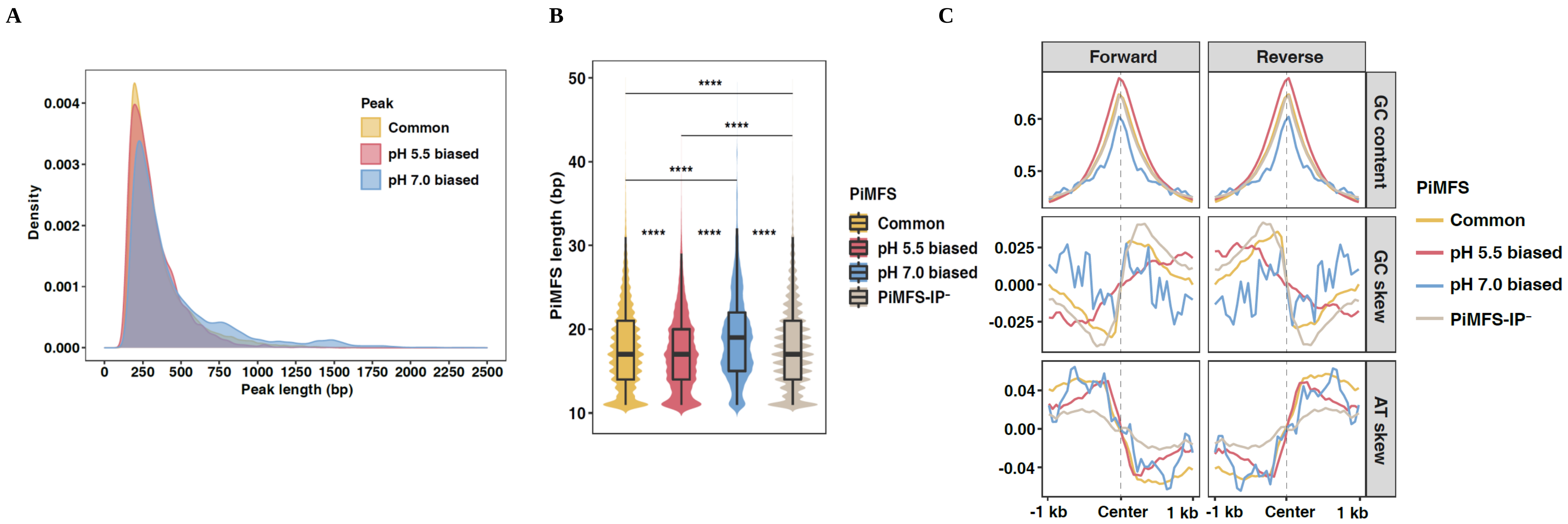

Figure S4 Characterization of the length, GC content and GC/AT skew of common, pH 5.5 and 7.0 biased iM peaks or PiMFSs-IP^{+}/IP^{-}(control). (**A**) Density of common, pH 5.5 and 7.0 biased iM peaks with different lengths. (**B**) The length of common, pH 5.5 and 7.0 biased PiMFSs-IP^{+} and PiMFSs-IP^{-}. Significance test was determined by using Wilcoxon rank-sum test. ** $p < 0.01$, **** $p < 0.0001$. **** between the two adjacent violin groups (the left panel) indicating significant difference between the two groups next to each other. (**C**) GC content and GC/AT skew of common, pH 5.5 and 7.0 biased PiMFSs-IP^{+} and PiMFSs-IP^{-}.

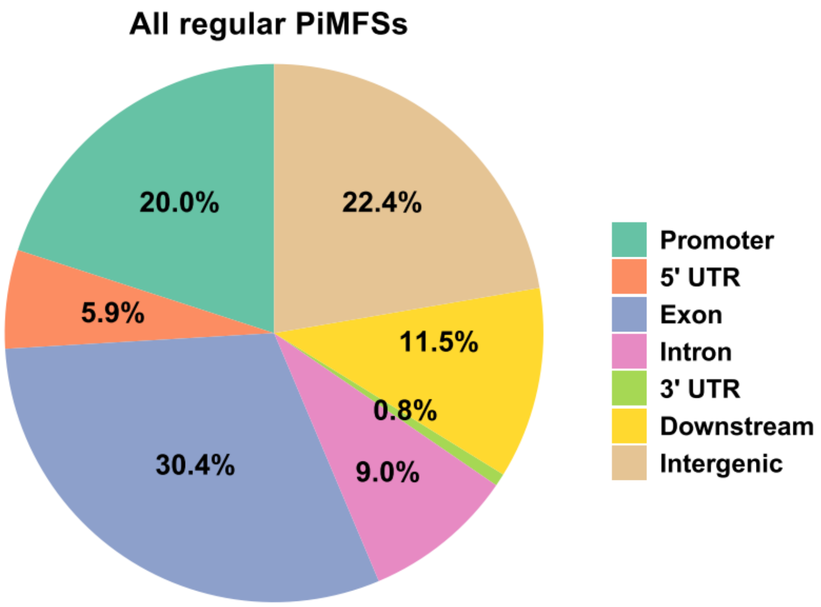

Figure S5 Subgenomic distributions of regular PiMFSs. The whole genome was partitioned into seven functionally annotated regions, including intergenic and downstream regions, 3’UTRs, introns, exons, 5’UTRs and promoters.

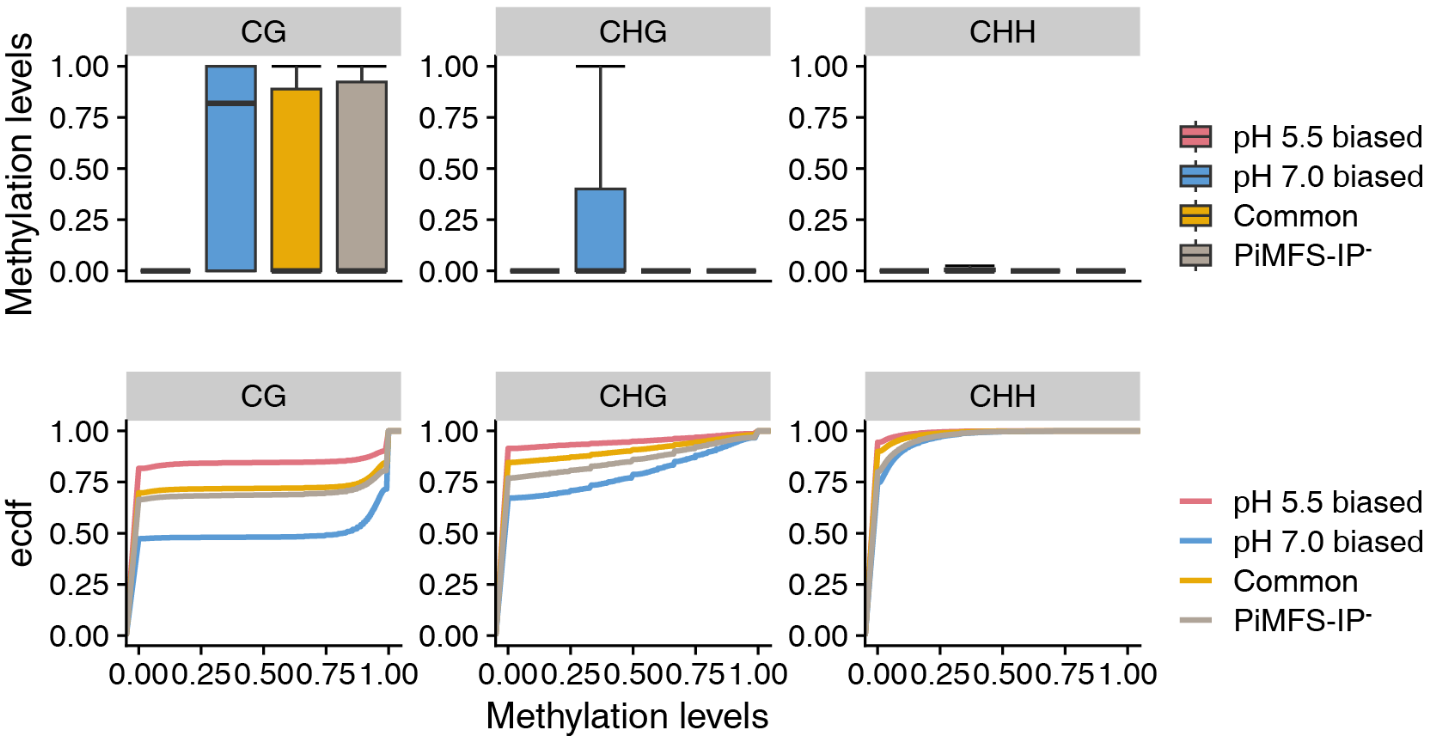

Figure S6 DNA methylation levels of PiMFSs in common, pH 5.5 and 7.0 biased PiMFSs-IP$^+$ peaks and PiMSs-IP$^-$. Boxplot (top) and cumulative curve (bottom) plot showing methylation levels of PiMFSs in common, pH 5.5 and 7.0 biased PiMFSs-IP$^+$ and PiMFSs-IP$^-$.

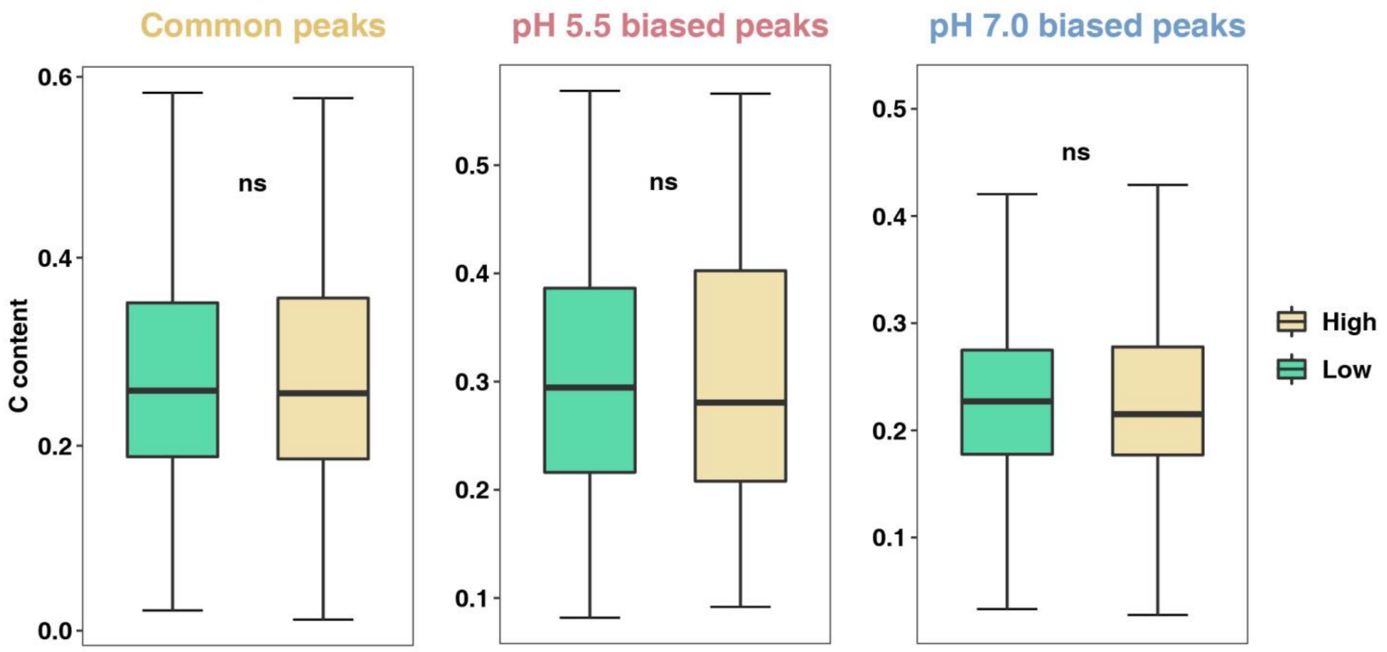

Figure S7 Normalization of C content for common, pH 5.5 and 7.0 biased iM peaks with high and low read density. Significance test was determined by using Wilcoxon rank-sum test, ns: no significance.

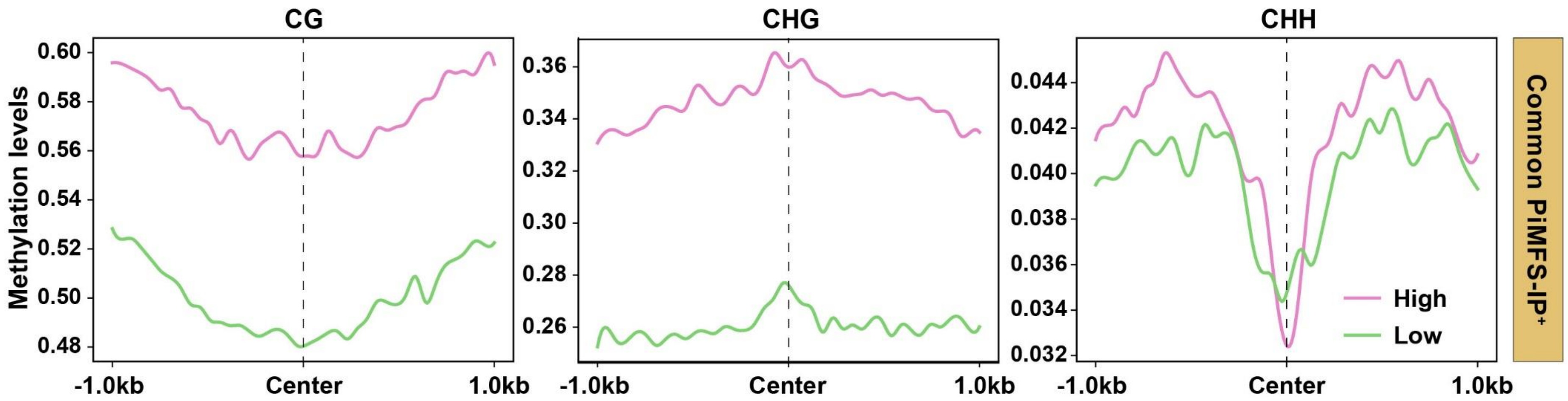

Figure S8 CG, CHG, and CHH methylation levels around ±1 kb of the center of common PiMFSs with similar C content between high and low read intensity of iM-IP-seq.

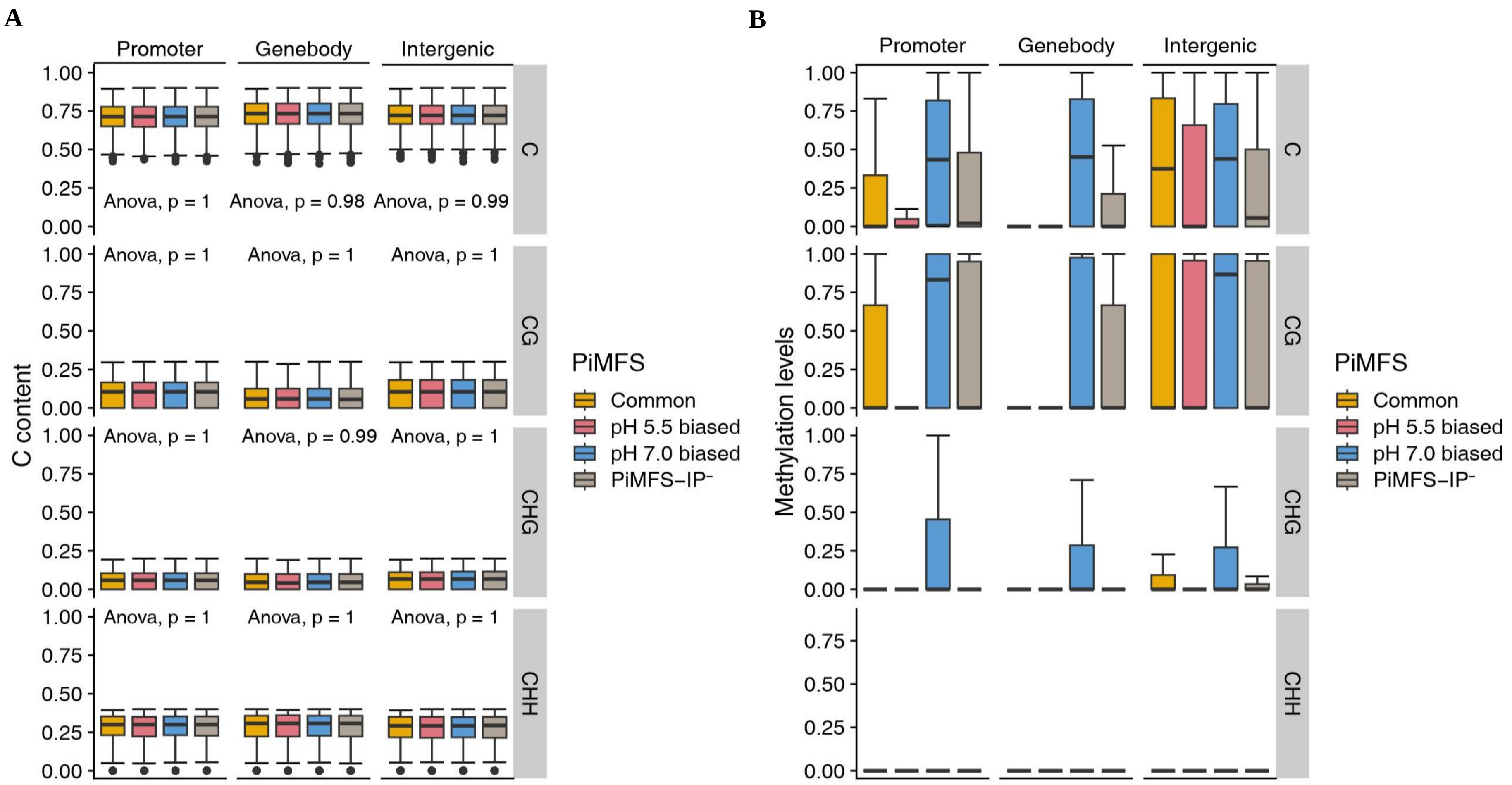

Figure S9 Normalization of total C, CG, CHG and CHH content for common, pH 5.5 and 7.0 biased iM peaks in different subgenomic regions. (A) Boxplots showing the content of total C, CG, CHG and CHH after normalization. Significance test was determined by using anova test. **(B)** Total C, CG, CHG, and CHH methylation levels for common (yellow), pH 5.5 (red) and 7.0 (blue) biased PiMFSs-IP$^+$ and PiMSs-IP$^-$ (grey) with similar C content, distributed in promoters, gene bodies and intergenic regions, respectively.

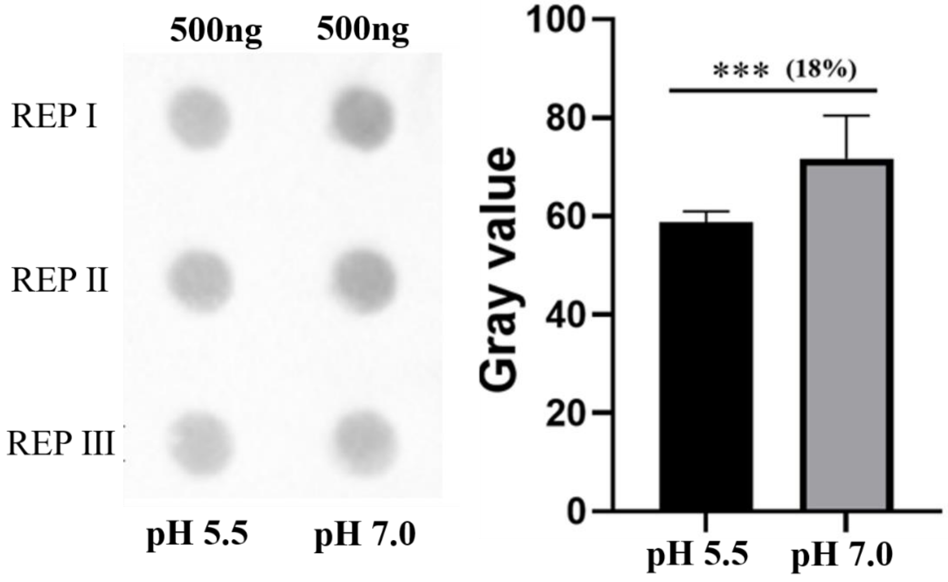

Figure S10 iMab based dot blotting detection of iM formation from genomic DNA reconstructed at pH 5.5 and 7.0 conditions. Significance test was determined by using One-way ANOVA analysis, *** $p < 0.001$.

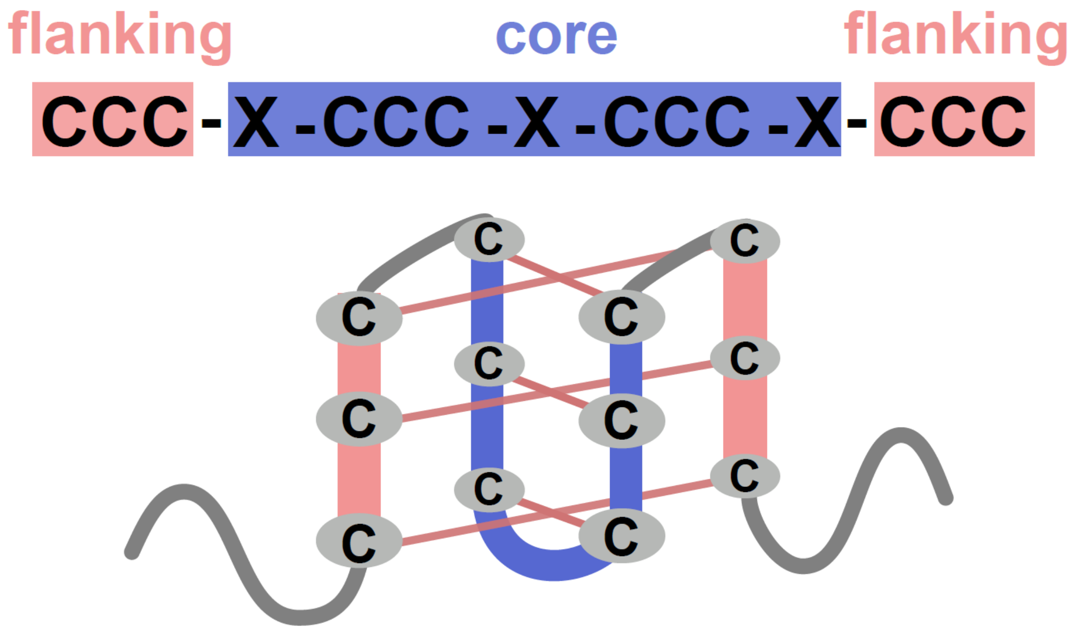

Figure S11 Diagram illustrating the core and flanking region of an iM with a PiMFS.

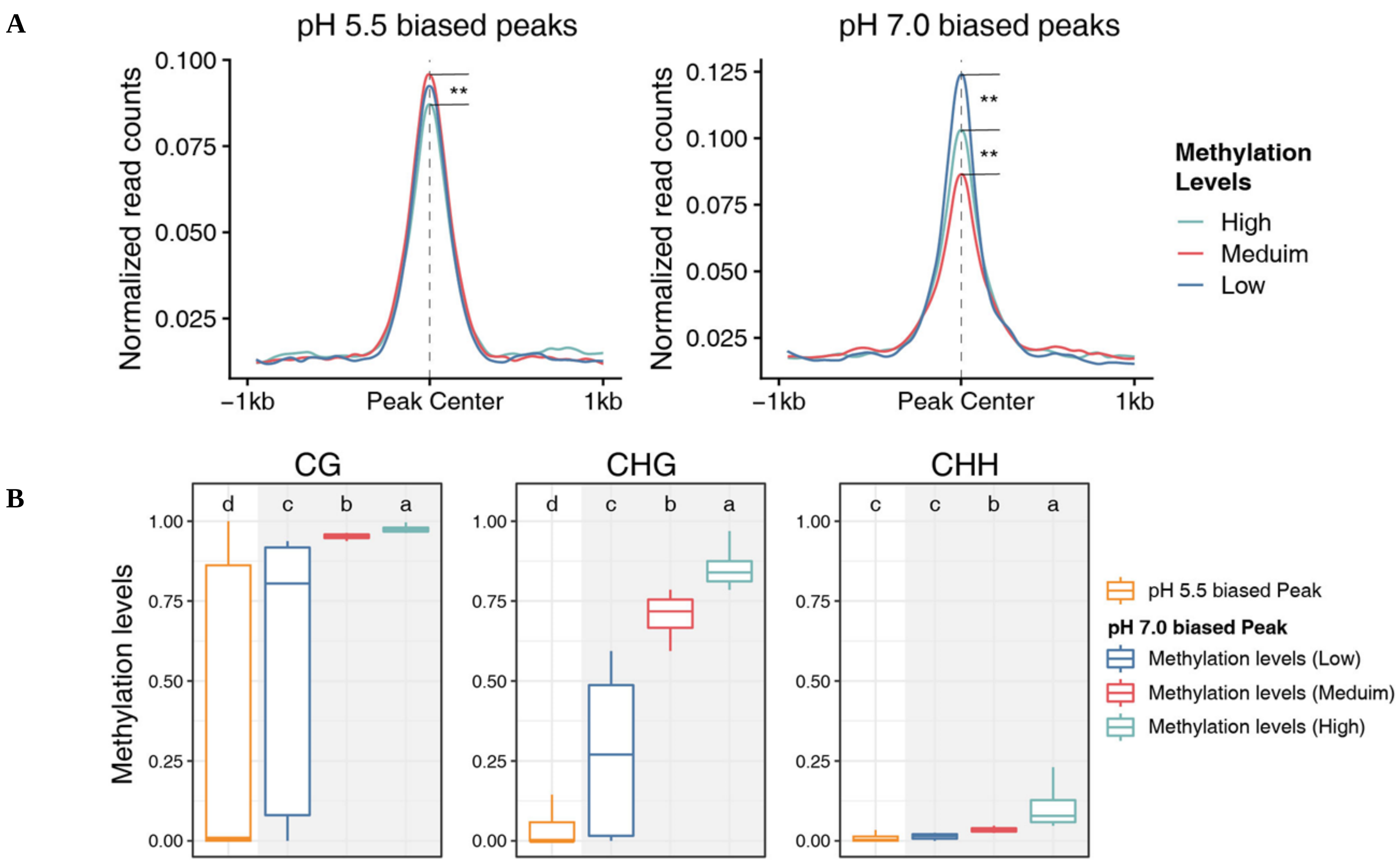

Figure S12 Relationship between DNA methylation levels and iM formation. (A) Normalized read counts of iM-IP-seq across ± 1 kb of the center of iM peaks with different DNA methylation levels (high, medium and low DNA methylation levels) but with equal peak numbers. Significance test was determined by using Wilcoxon rank-sum test. ** $p < 0.01$. **(B)** Methylation levels of CG, CHG, and CHH for pH 5.5 biased peaks and three subtypes of pH 7.0 biased peaks as indicated in Figure S12A. Significance test was determined by using ANOVA test. Different letters between any of two groups indicating significant difference.

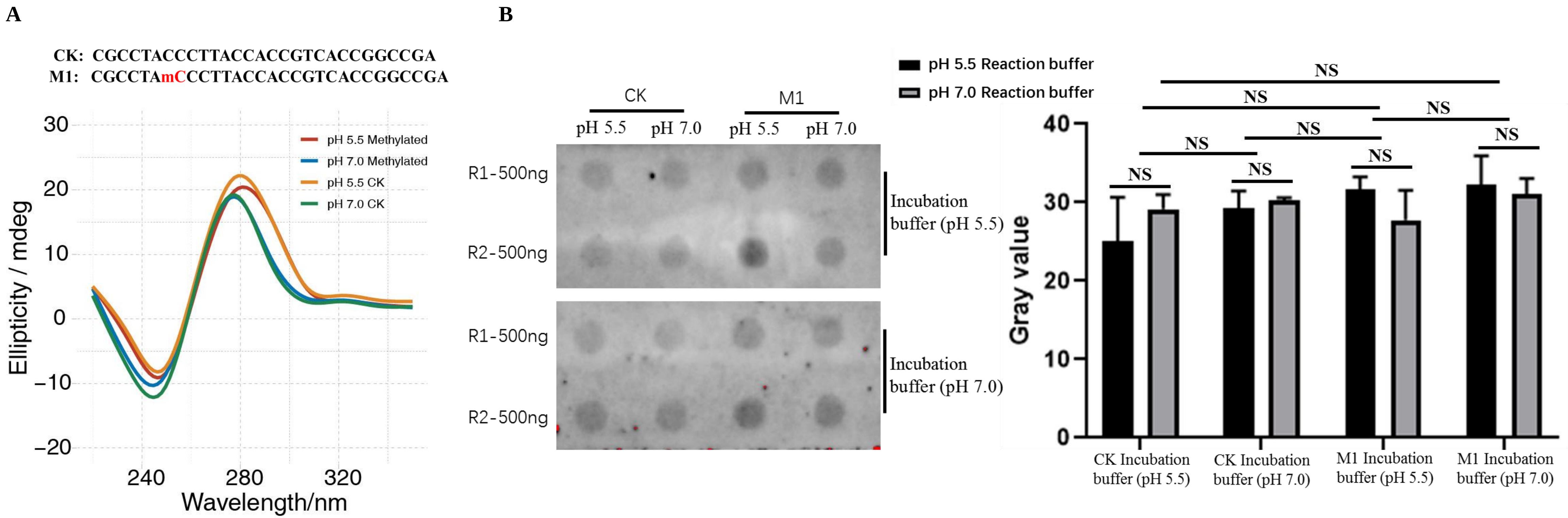

Figure S13 CD and iMab based dot blotting assays for CK and methylated (M1) DNA oligoes at pH 5.5 and 7.0 conditions, respectively. (**A**) CD detection for CK and M1 DNA oligoes under pH 5.5 and 7.0 conditions, respectively. (**B**) Dot blotting assays for CK and M1 DNA oligoes under pH 5.5 and 7.0 conditions, respectively. Significance test was determined by using One-way ANOVA analysis, *** $p < 0.001$, ** $p < 0.01$, * $p < 0.05$.

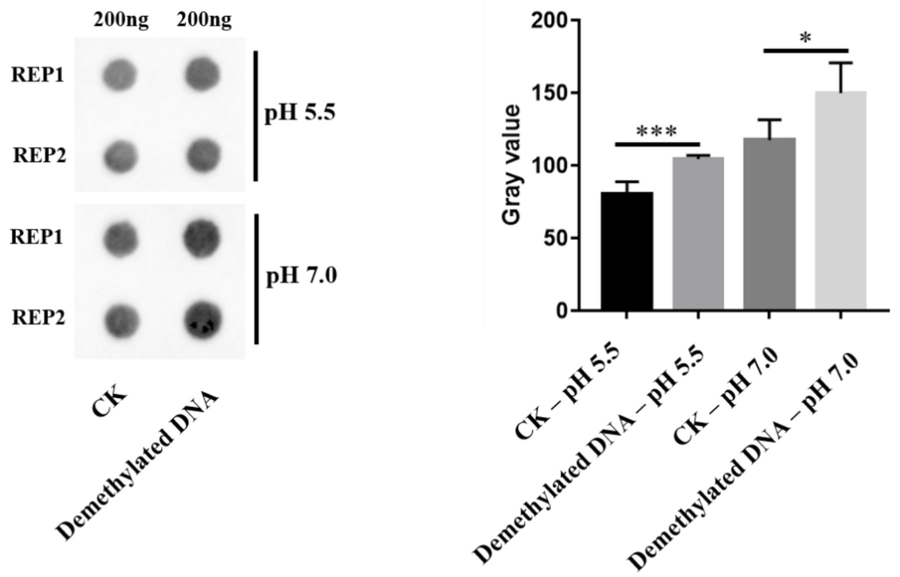

Figure S14 iMab based dot blotting assays using demethylated and CK genomic DNA at pH 5.5 and 7.0 conditions.

Significance test was determined by using One-way ANOVA analysis, *** $p < 0.001$, ** $p < 0.01$, * $p < 0.05$.

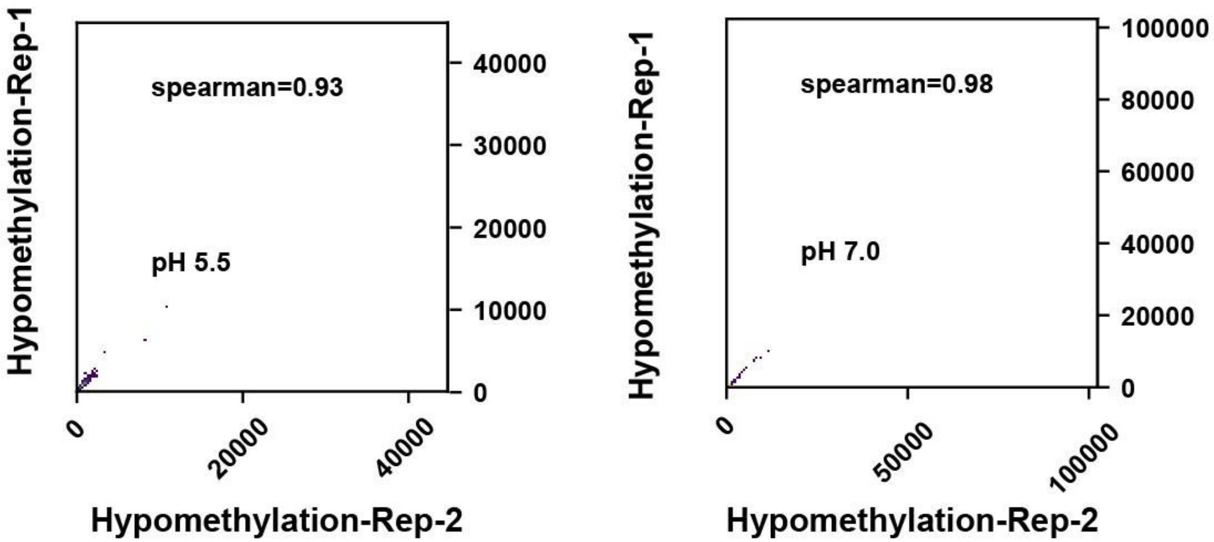

Figure S15 Correlation analyses of two biologically replicated iM-IP-seq data sets using Hypomethylated DNA at pH 5.5 and 7.0 conditions.

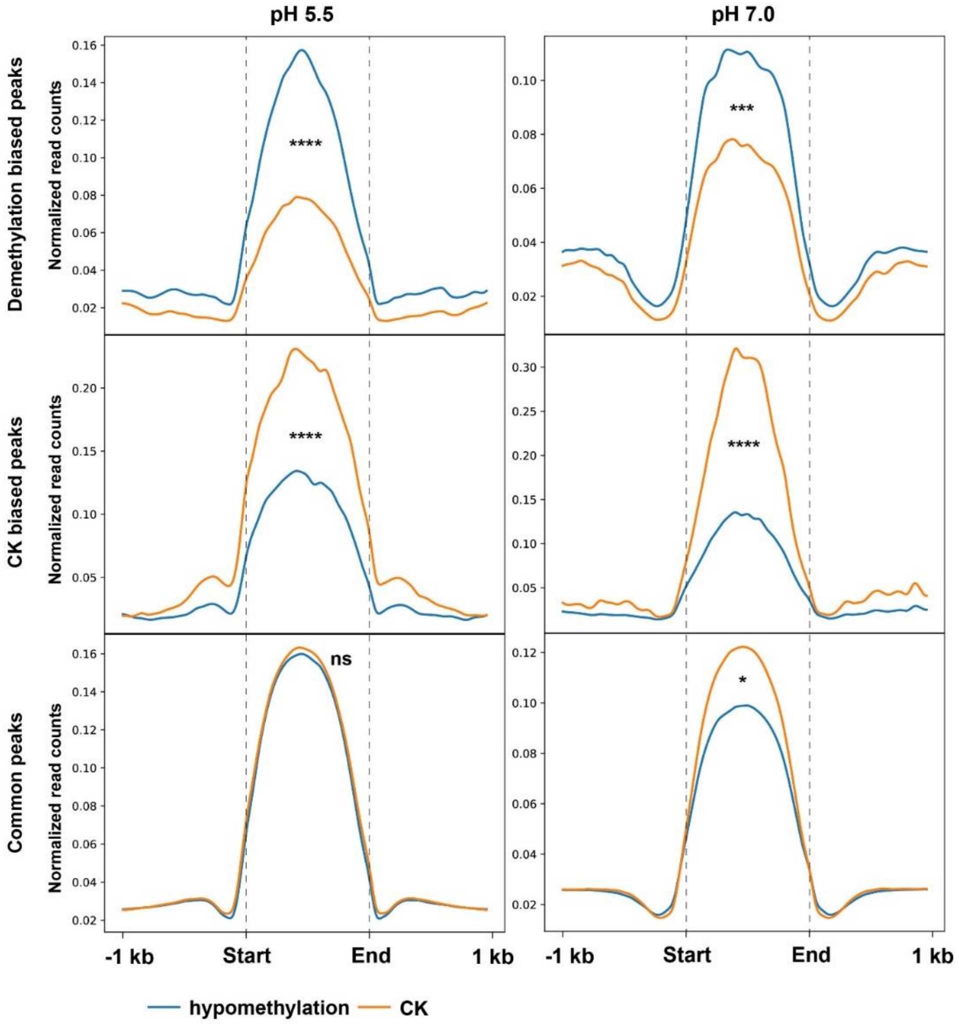

Figure S16 Normalized iM-IP-seq read counts distributed around ±1 kb from the start to the end of common, CK and hypomethylation biased iM peaks at pH 5.5 and 7.0 conditions. Significance test was determined by using Wilcoxon rank-sum test. * $p < 0.05$, *** $p < 0.001$, **** $p < 0.0001$, ns: no significance.

P1F-CG	ATATAAATTACGACGAATCGACGTAATATAACGTAGATTATT
P1R-CG	TATATTTAATGCTGCTTAGCTGCATTATATTGCATCTAATAA
P2F-CHG	ATATAAATTACTGACTGAATCAGACAGTAATATAACTGTAGATTATT
P2R-CHG	TATATTTAATGACTGACTTAGTCTGTCATTATATTGACATCTAATAA
P3F-CHH	ATATAAATTACTACAAATCTACTTAATATAACTTAGATTATT
P3R-CHH	TATATTTAATGATGTTTAGATGAATTATATTGAATCTAATAA

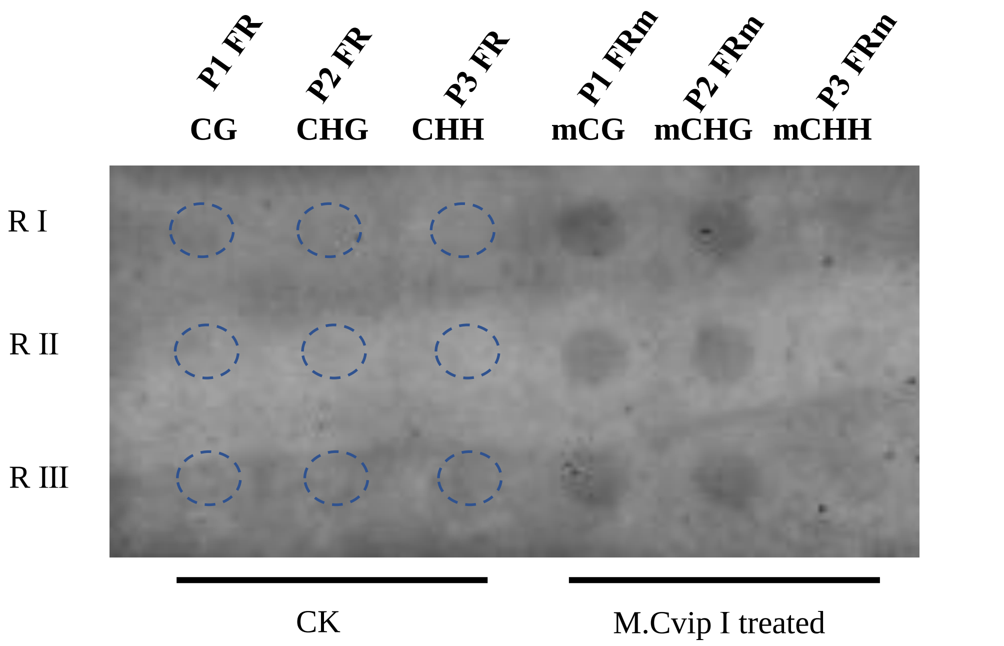

Figure S17 Anti-5mC antibody based dot blotting for synthesized oligoes containing only CG, CHG and CHH, respectively, treated with or without (CK) M.Cvip I. The top panel showing sequence information containing CG, CHG or CHH only for annealing oligo dimer for M.Cvip I treatment.

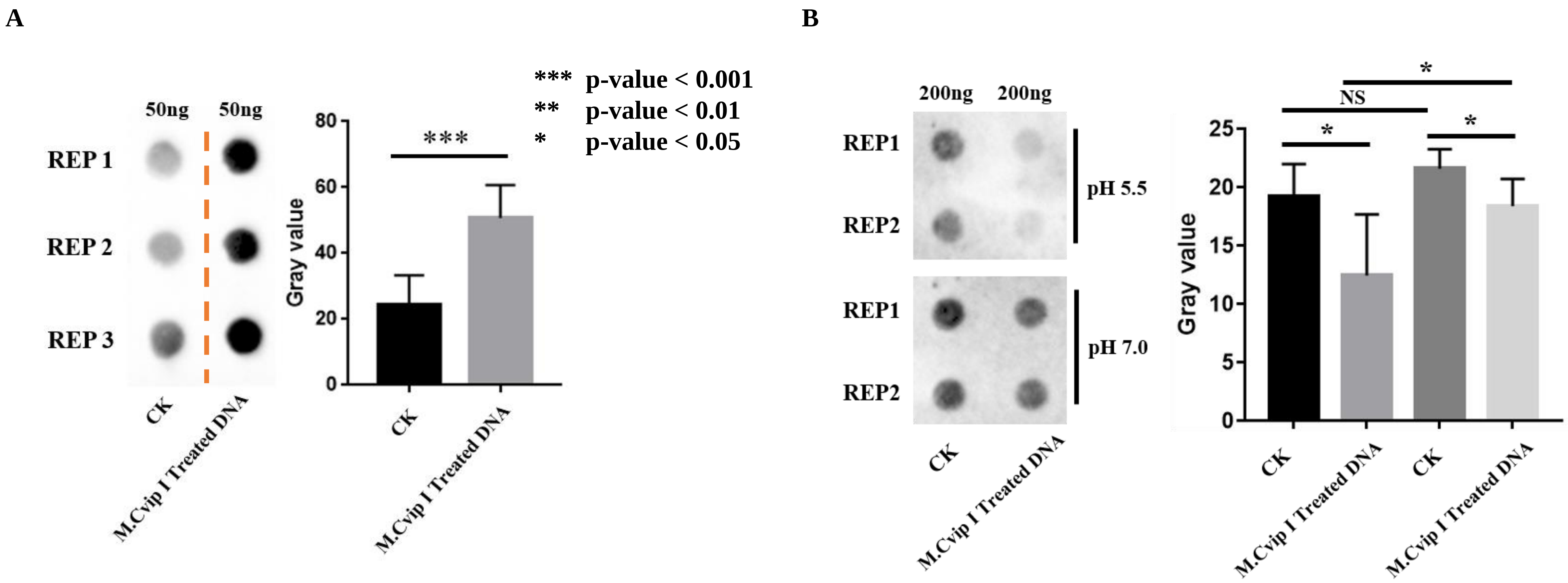

Figure S18 Dot blotting detection of DNA methylation and i-motifs using M.Cvip I treated and CK genomic DNA. (**A**) Anti-5mC antibody based dot blotting detection of DNA methylation between M.Cvip I treated and CK genomic DNA. (**B**) iMab based dot blotting detection of iMs between M.Cvip I treated and CK genomic DNA under pH 5.5 and 7.0 conditions. Significance test was determined by using One-way ANOVA analysis, *** $p < 0.001$, ** $p < 0.01$, * $p < 0.05$.

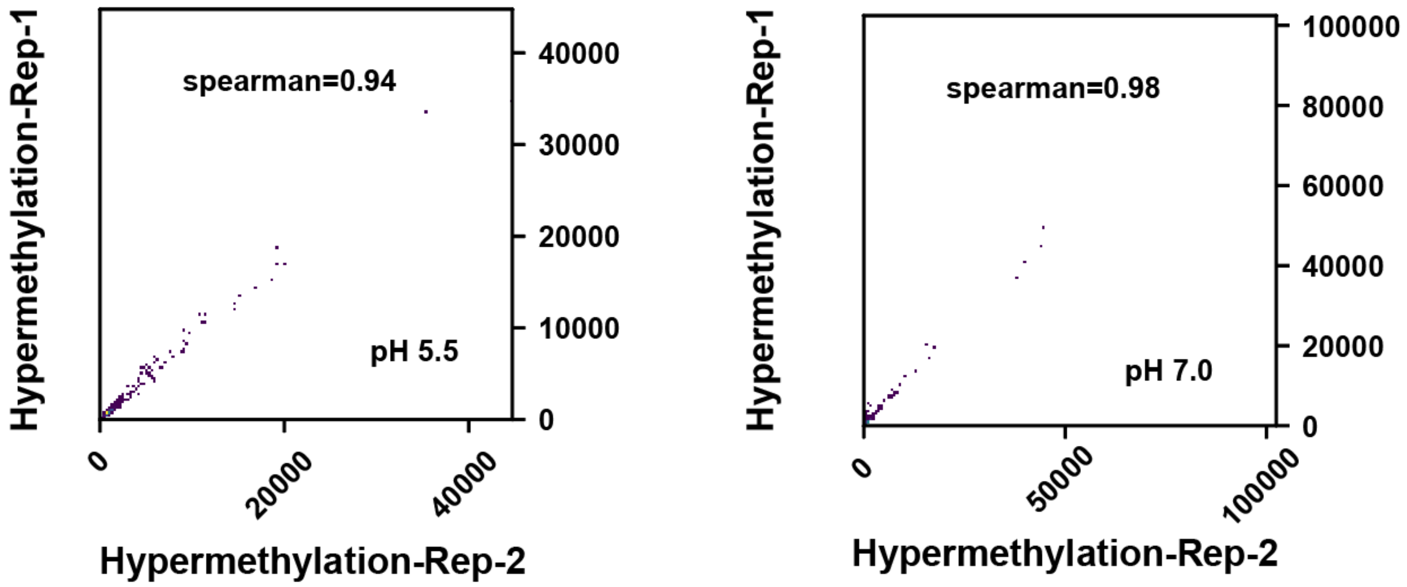

Figure S19 Correlation analyses of two biologically replicated iM-IP-seq data sets using M.Cvip I treated (hypermethylation) DNA at pH 5.5 and 7.0 conditions.

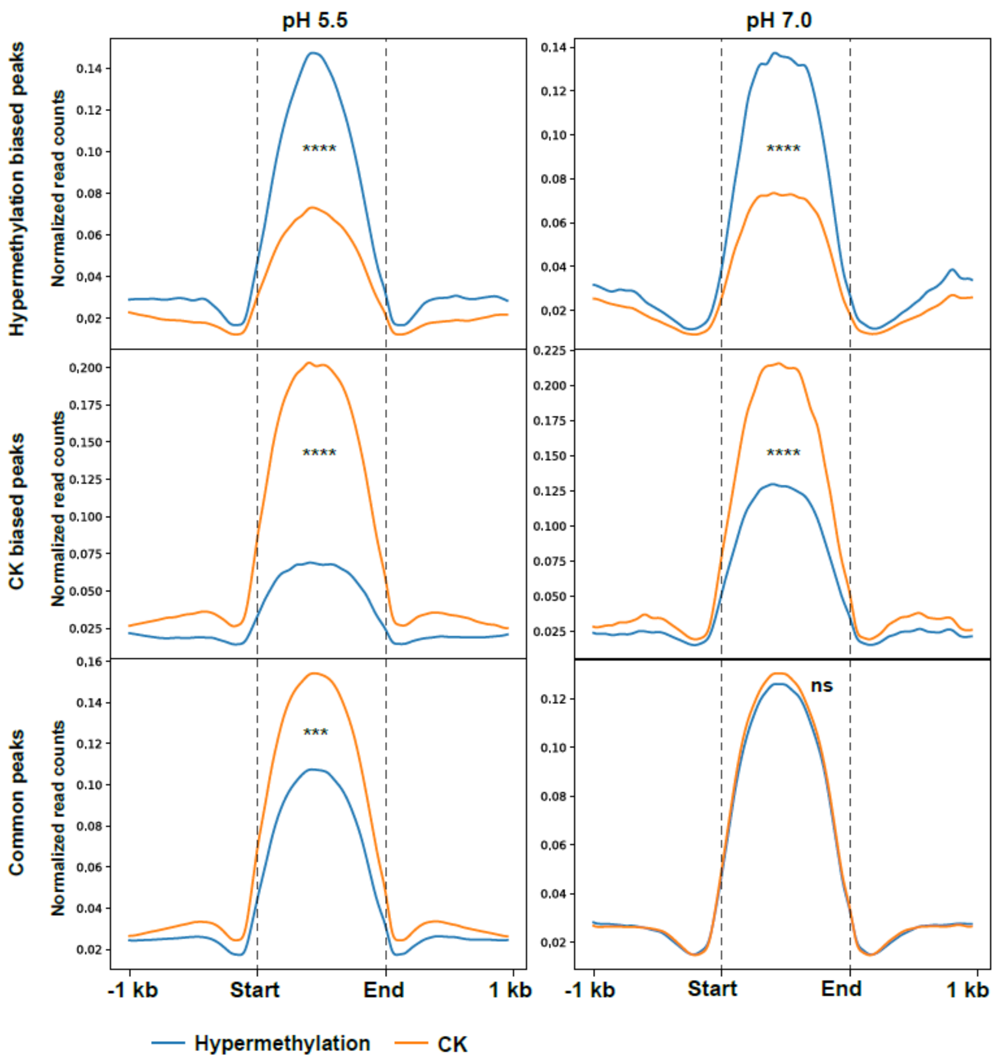

Figure S20 Normalized iM-IP-seq read counts distributed around ±1 kb from the start to the end of common, CK and hypermethylation biased iM peaks at pH 5.5 and 7.0 conditions. Significance test was determined by using Wilcoxon rank-sum test. *** $p < 0.001$, **** $p < 0.0001$, ns: no significance.

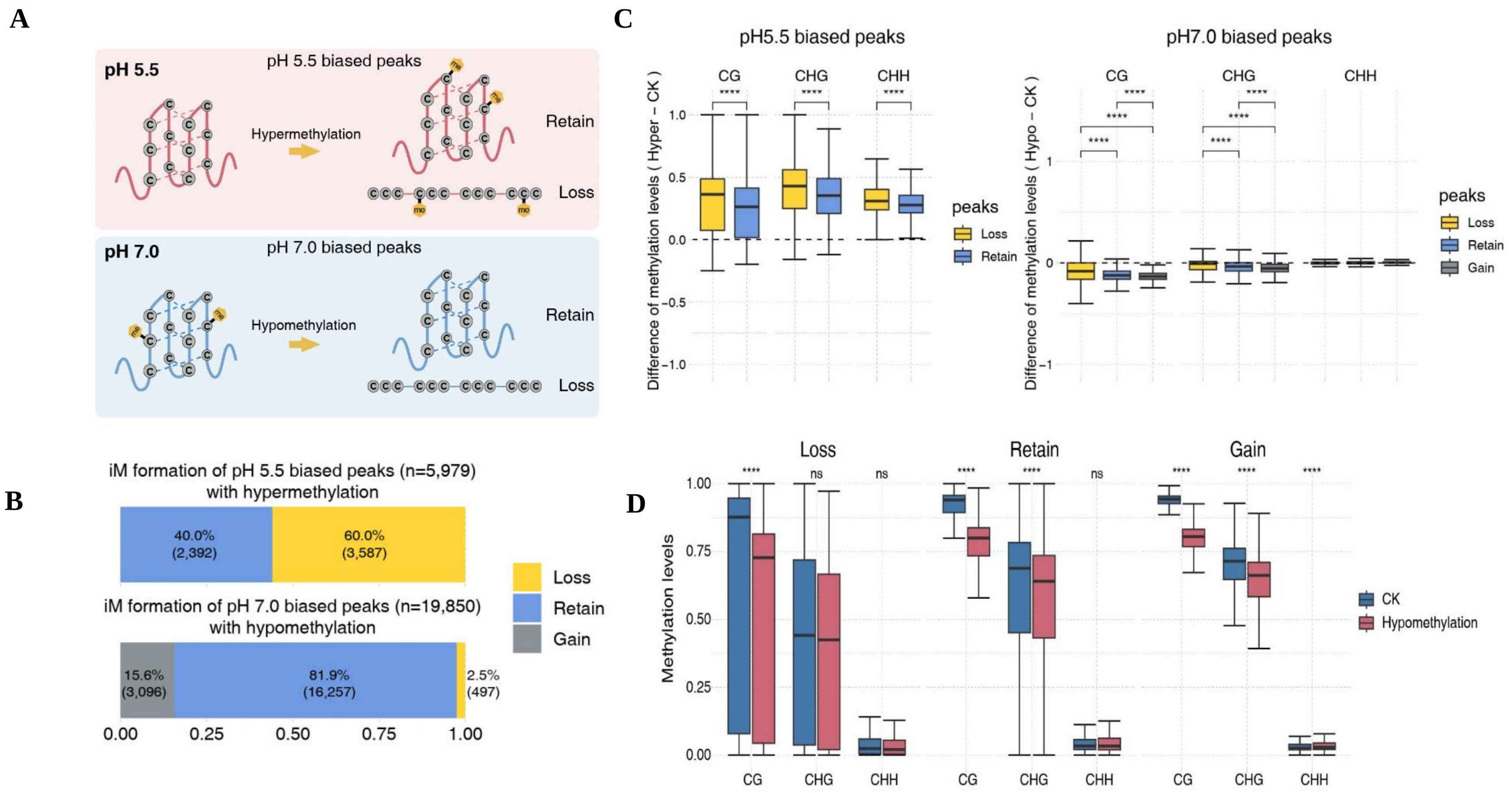

Figure S21 DNA methylation changes of hyper or hypo methylation related dynamics of pH 5.5 and 7.0 biased iMs at pH 5.5 and 7.0, respectively. (A) Diagram showing hyper or hypo methylation related dynamic iMs of pH 5.5 and 7.0 biased iMs at pH 5.5 (top) and 7.0 (bottom), respectively. **(B)** Barplot showing number and percentage of hyper or hypo methylation related loss, retain and gain of pH 5.5 and 7.0 biased iMs at pH 5.5 (top) and 7.0 (bottom), respectively. **(C)** Hyper (left) or hypo (right) methylation related changes of CG, CHG, and CHH methylation levels relative to CK for three subtypes of dynamic iMs as shown in Figure S21B. Significance test was determined by using Wilcoxon rank-sum test. * $p < 0.05$, ** $p < 0.01$, **** $p < 0.0001$. **(D)** CG, CHG, and CHH methylation levels for three subtypes of hypomethylation related dynamic pH 7.0 biased peaks relative to CK. Significance test was determined by using Wilcoxon rank-sum test. **** $p < 0.0001$.

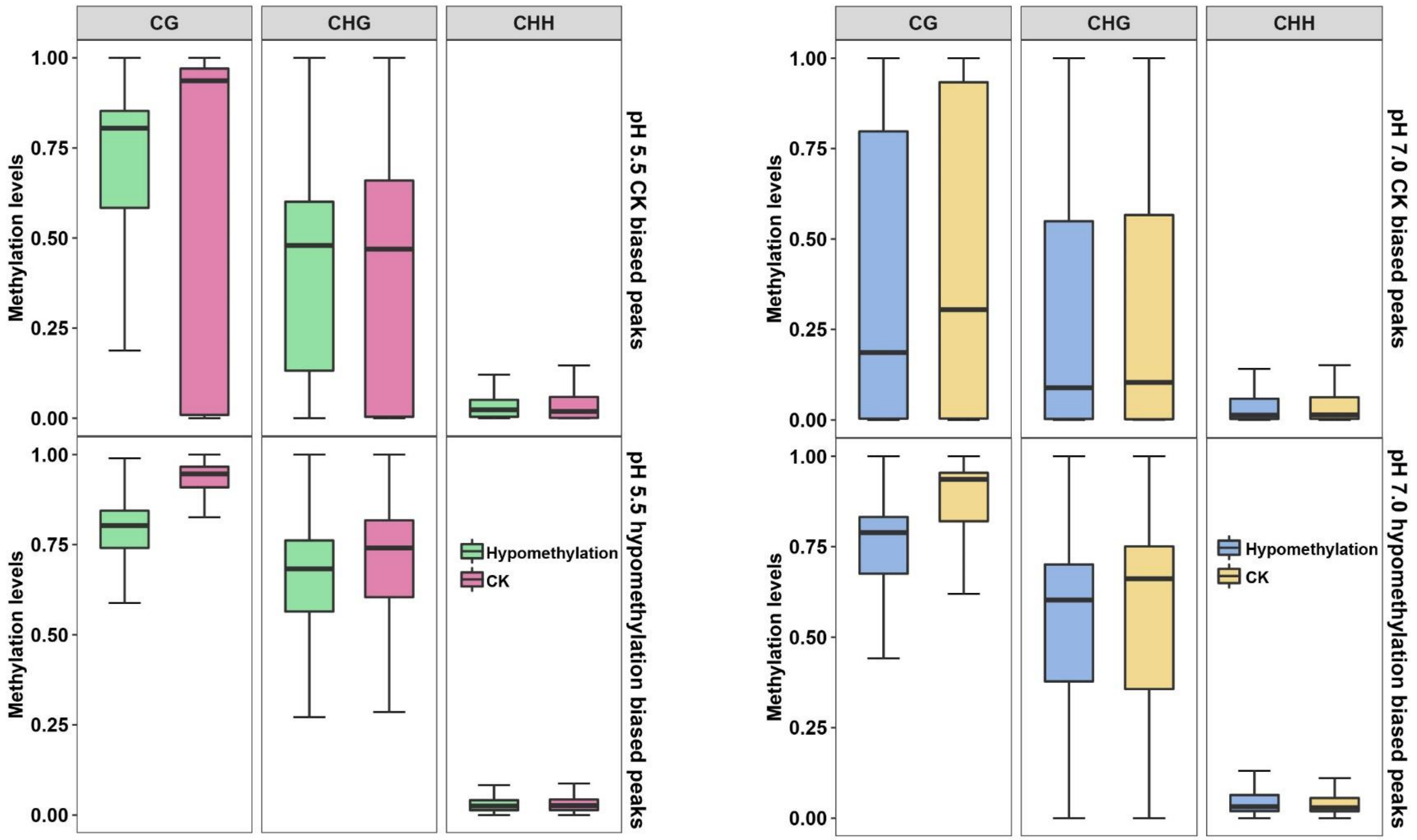

Figure S22 Box blot showing CG/CHG/CHH methylation levels of CK and hypomethylation biased peaks at pH 5.5 (left) and pH 7.0 (right) conditions.

Significance test was determined by using Wilcoxon rank-sum test. * $p < 0.05$, ** $p < 0.01$, *** $p < 0.001$, **** $p < 0.0001$.

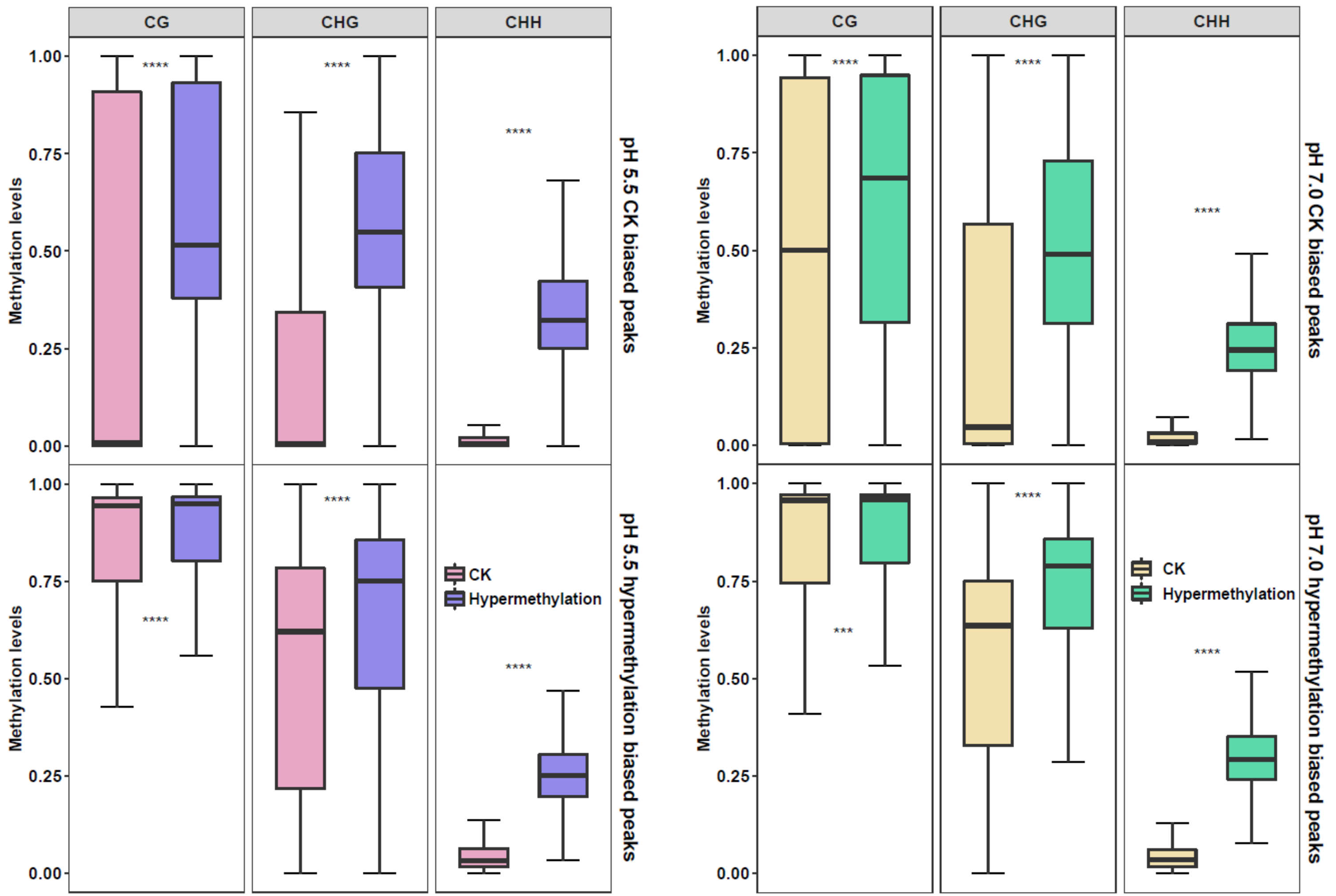

Figure S23 Box blot showing CG/CHG/CHH methylation levels of CK and hypermethylation biased peaks at pH 5.5 (left) and pH 7.0 (right) conditions. Significance test was determined by using Wilcoxon rank-sum test. *** $p < 0.001$, **** $p < 0.0001$.

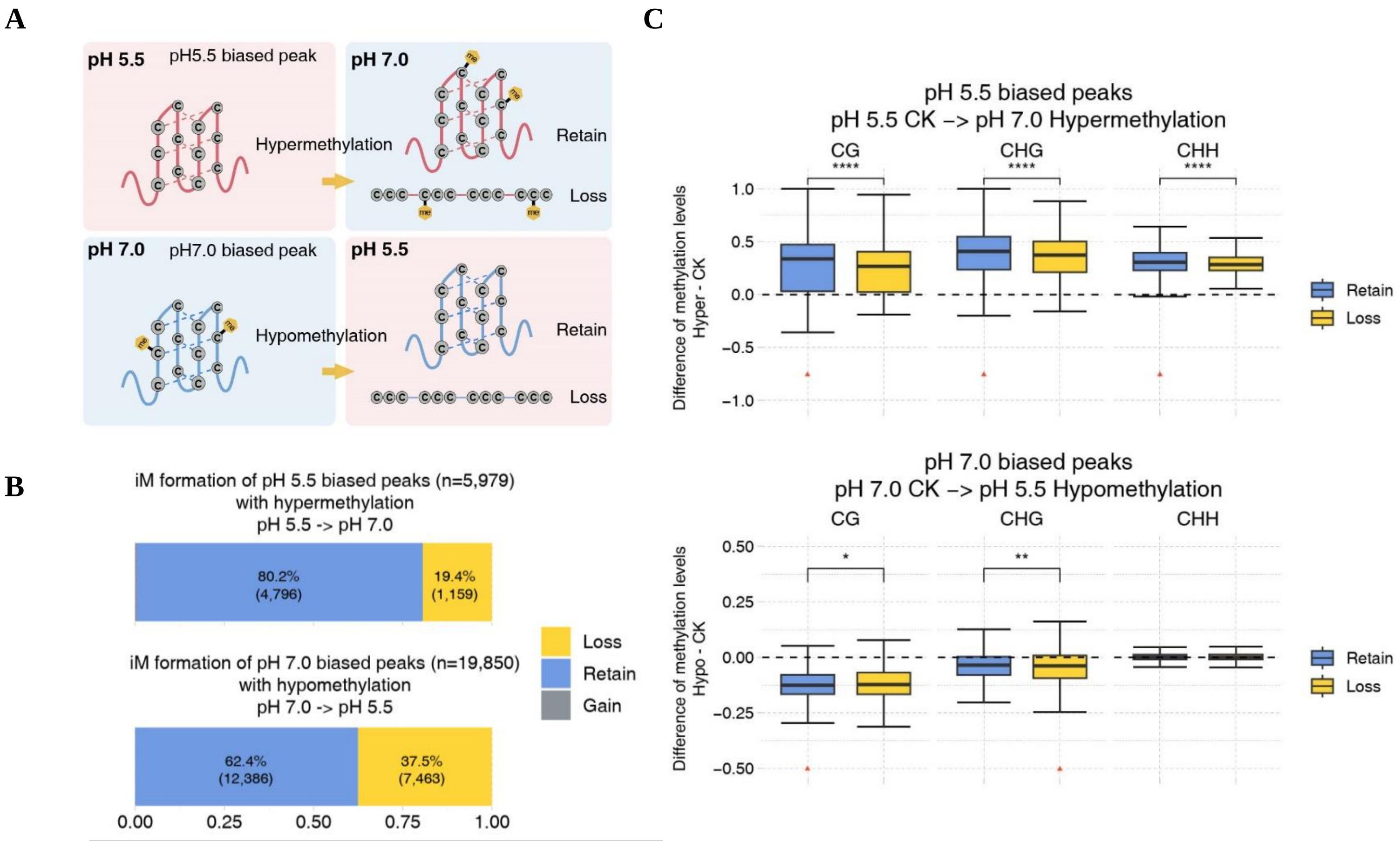

Figure S24 DNA methylation changes of hyper or hypo methylation related dynamics of pH 5.5 and 7.0 biased iMs at pH 7.0 and 5.5, respectively. (A) Diagram showing hyper or hypo methylation related dynamics of pH 5.5 and 7.0 biased iMs at pH 7.0 (top) and 5.5 (bottom), respectively. **(B)** Barplot showing number and percentage of hyper or hypo methylation related loss, retain and gain of pH 5.5 and 7.0 biased iMs at pH 7.0 (top) and 5.5 (bottom), respectively. **(C)** Hyper (top) or hypo (bottom) methylation related changes of CG, CHG, and CHH methylation levels relative to CK for retain and loss of iMs as shown in Figure S24B. Significance test was determined by using Wilcoxon rank-sum test. * $p < 0.05$, ** $p < 0.01$, **** $p < 0.0001$. The red diamonds under each box indicating the group with a larger average difference value.

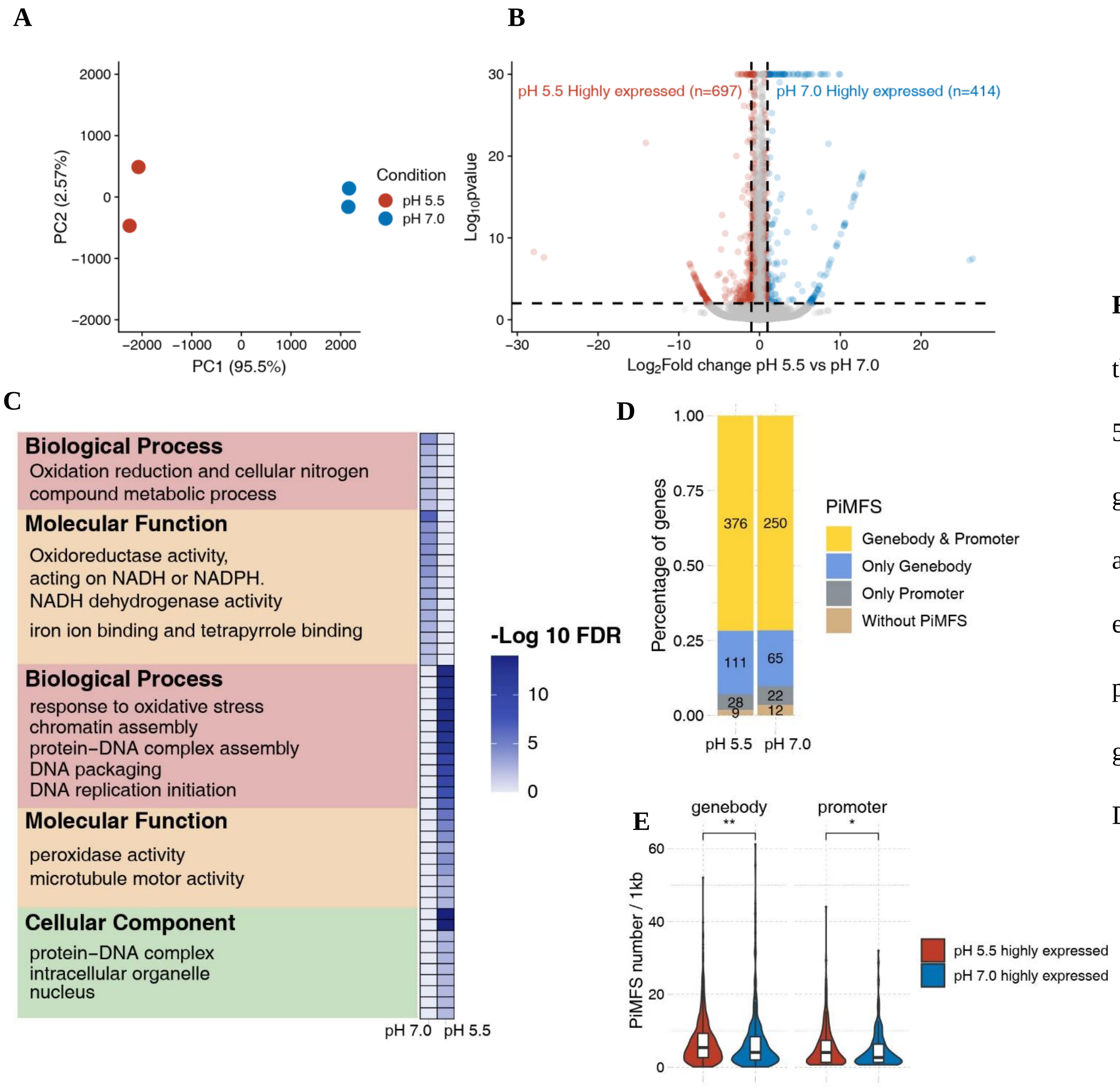

Figure S25 Analyses of pH dependent transcriptome. (A) Scatter plot showing the Principal Component Analysis for RNA-seq from seedlings grown under pH 5.5 and 7.0 conditions. **(B)** Volcano plot showing the differentially expressed genes (DEGs) under two pH, 667 and 414 genes highly expressed under pH 5.5 and 7.0, respectively. **(C)** Heatmap showing the –log 10 FDR of GO terms enriched in highly expressed genes under pH 5.5 and 7.0, respectively. (**D**) Bar plot showing the number of genes for each group associated with PiMFSs in genic regions. (**E**) Violin plot showing the density of PiMFSs in genic regions for DEGs under pH 5.5 and 7.0. ** $p < 0.01$, * $p < 0.05$.

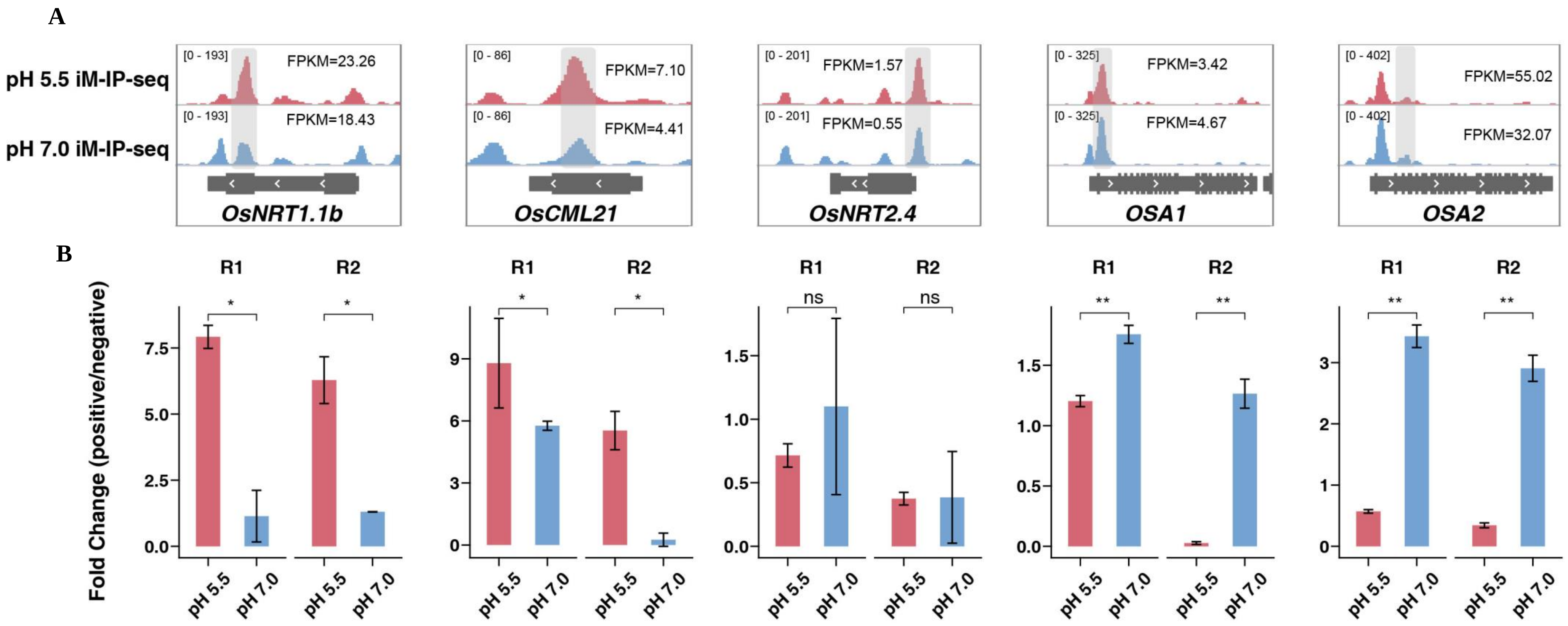

Figure S26 iMab related CUT&TAG qPCR assays for pH responsive genes. (A) IGV snapshot showing 5 functional pH responsive genes, including *OsNRT1.1B*, *OsNRT2.4*, *OSA1*, *OSA2* and *OsCML21*, with differential enrichment levels of *in vitro* iMs under pH 5.5 and 7.0 conditions, FPKM values were calculated from transcriptome data under different pH, the grey rectangle marked in the genic region for designing primers for CUT&TAG qPCR assays. **(B)** Bar plot showing the CUT&TAG qPCR enrichment levels of *in vivo* iMs within the aforementioned 5 functional pH responsive genes. Two independent biological replicates were listed for showing a similar trend of iM changes between pH 5.5 and 7.0. ** $p < 0.01$, * $p < 0.05$, ns: no significance.

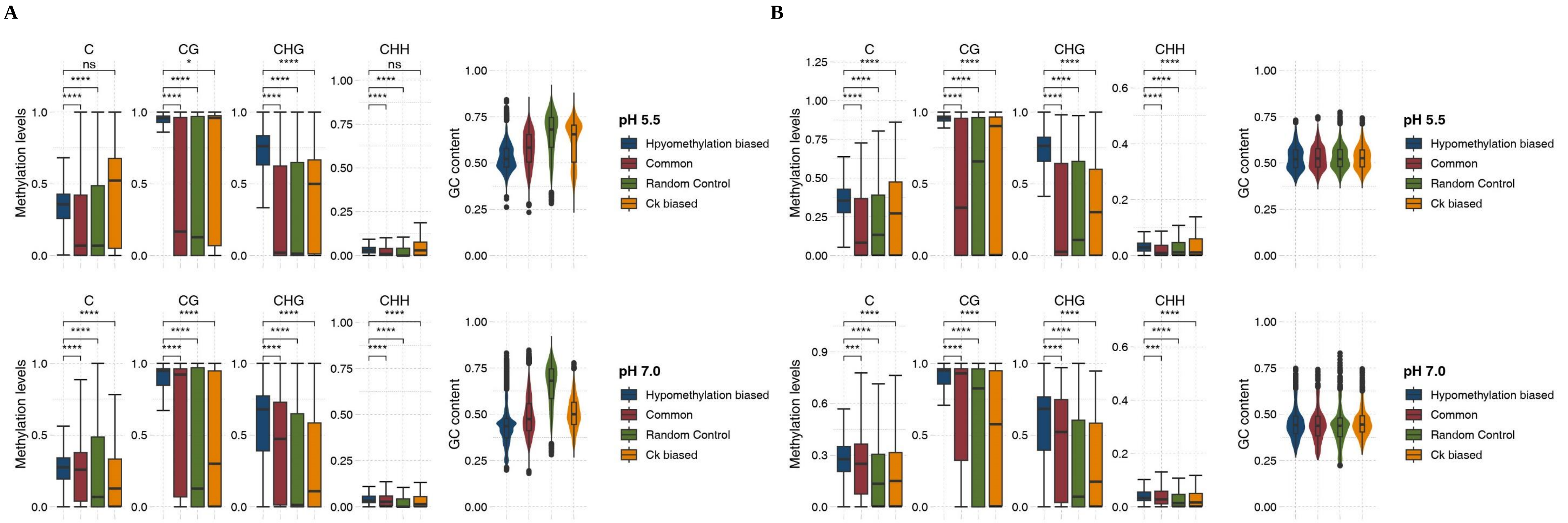

Figure S27 Box blot showing C/CG/CHG/CHH methylation levels of CK and hypomethylation biased peaks at pH 5.5 (top) and 7.0 (bottom) conditions. (A) The primary result for comparisons of DNA methylation levels in each cytosine context**. (B)** Each group was normalized to similar GC content for comparisons of DNA methylation levels in each cytosine context**.** Significance test was determined by using Wilcoxon rank-sum test. *** $p < 0.001$, **** $p < 0.0001$, ns non-significant. Violin plot showing the GC content among each group.

Table S1 Number of PiMFSs associated with *in vitro* iMs identified at pH 5.5 and 7.0 conditions

	No. of peaks	No. of PiMFSs	Percentage of *in vitro* iMs peaks with PiMFSs
pH 5.5 specific	33,455	290,015	87.4%
pH 7.0 specific	55,062	326,373	72.3%
Common	18,877/19,150	199,579/150,736	90.5%/88.5%

Table S2 Number of PiMFSs at pH 5.5 and 7.0 conditions

	No. of peaks	No. of PiMFSs	Percentage of *in vitro* iMs peaks with PiMFSs
pH 5.5 biased	5,979	55,778	87.5%
pH 7.0 biased	19,850	72,649	60.9%
Common	39,179	329,443	83.0%

Table S3 i-motifs or PiMFSs related to hyper/de-methylation at pH 5.5 and 7.0 conditions

		No. of Peaks	No. of PiMFSs
pH 5.5 demethylation vs CK	demethylation biased	8,592	21,150
	CK biased	1,721	17,646
	common	72,646	587,002
pH 7.0 demethylation vs CK	demethylation biased	6,902	45,289
	CK biased	2,420	15,678
	common	70,727	522,369
pH 5.5 hypermethylation vs CK	hypermethylation biased	43,280	117,176
	CK biased	7,290	95,847
	common	44,283	333,843
pH 7.0 hypermethylation vs CK	hypermethylation biased	9,445	146,572
	CK biased	4,170	21,937
	common	61,851	573,479

Table S4 Number of differential methylation regions (DMRs)

	C Context	Hypermethylation	Hypomethylation
**DMRs**	CG	115,672	182,125
	CHG	283,720	3,868
	CHH	656,623	3,890

$p < 0.01$ & $\Delta mC \geq 0.1$
